# Retrospective analysis of enhancer activity and transcriptome history

**DOI:** 10.1038/s41587-023-01683-1

**Published:** 2023-02-23

**Authors:** Ruben Boers, Joachim Boers, Beatrice Tan, Marieke E. van Leeuwen, Evelyne Wassenaar, Erlantz Gonzalez Sanchez, Esther Sleddens, Yasha Tenhagen, Eskeatnaf Mulugeta, Joop Laven, Menno Creyghton, Willy Baarends, Wilfred F. J. van IJcken, Joost Gribnau

**Affiliations:** 1 Department of Developmental Biology, Erasmus University Medical Center Rotterdam, Rotterdam, Netherlands; 2 Department of Cell Biology, Erasmus University Medical Center Rotterdam, Rotterdam, Netherlands; 3 Department of Obstetrics and Gynaecology, Erasmus University Medical Center Rotterdam, Rotterdam, Netherlands; 4 Erasmus Center for Biomics, Erasmus University Medical Center Rotterdam, Rotterdam, Netherlands

**Keywords:** Transcriptomics, Assay systems, Stem-cell differentiation, Time series

## Abstract

Cell state changes in development and disease are controlled by gene regulatory networks, the dynamics of which are difficult to track in real time. In this study, we used an inducible DCM–RNA polymerase subunit b fusion protein which labels active genes and enhancers with a bacterial methylation mark that does not affect gene transcription and is propagated in S-phase. This DCM–RNA polymerase fusion protein enables transcribed genes and active enhancers to be tagged and then examined at later stages of development or differentiation. We apply this DCM-time machine (DCM-TM) technology to study intestinal homeostasis, revealing rapid and coordinated activation of enhancers and nearby genes during enterocyte differentiation. We provide new insights in absorptive–secretory lineage decision-making in intestinal stem cell (ISC) differentiation and show that ISCs retain a unique chromatin landscape required to maintain ISC identity and delineate future expression of differentiation-associated genes. DCM-TM has wide applicability in tracking cell states, providing new insights in the regulatory networks underlying cell state changes.

## Abstract (web summary)

Labeling transcribed genes and active enhancers with a bacterial methylation mark traces cell state changes.

## Main

Embryonic development and cell differentiation are intricate processes directed by crosstalk between cells that affect cell fate decisions and the establishment of cell-type-specific gene expression programs^1–3^. Lineage tracing studies have been crucial to understand these processes. Initial studies applied light microscopy to follow cleavage divisions, and, more recently, barcoding, cre-lox and other genetic systems have been used to mark precursors or progenitors for readout at later stages of development or differentiation^4^. The present advance of single-cell RNA sequencing (scRNA-seq) technologies provides a wealth of expression data that can be used to predict developmental trajectories in silico and can be linked to genetic lineage-tracing techniques to rebuild lineage trees^5–7^.

Application of these tracing technologies to study the epithelium of the small intestine provided critical insights in homeostasis and regeneration. Turnover of this epithelium happens within 7 days and starts with division of the intestinal stem cell (ISC) located at the bottom of the intestinal crypt^8^. ISCs give rise to progenitors that divide moving up the intestinal crypt, meanwhile committing to the absorptive or secretory lineage. Absorptive progenitors mature into enterocytes, whereas secretory progenitors give rise to Paneth, tuft, enteroendocrine and goblet cells. ISCs are flanked by Paneth cells that provide Wnt, Notch and epidermal growth factor (EGF) signals required for self-renewal. Loss of ISC–Paneth cell contact facilitates cell differentiation, aided by bone morphogenetic protein (BMP) signaling that further supports maturation of differentiated cell types. Notch signaling also plays a crucial role in lineage commitment remaining high in absorptive progenitors and is downregulated in secretory progenitors. Lineage-tracing and scRNA-seq experiments have been instrumental in identification and characterization of the crypt-based columnar cell as the ISC^9^ but also showed that several other cell types, including enteroendocrine, Paneth and immature enterocytes, provide a reservoir of cells that can replenish the ISC niche in injury-induced regeneration^10–12^.

Although these examples highlight the successful application of lineage-tracing and scRNA-seq technologies to build relationships between cellular trajectories, they cannot keep track of cell state changes following this trajectory and provide limited depth and temporal information with respect to gene expression changes^13^. To facilitate whole-genome cell state tracing, we developed a system to tag transcribed genes with DCM methylation labels to be examined at later stages of development or differentiation. We made use of a fusion between DCM and RNA polymerase 2 subunit b to DCM-label gene bodies of transcribed genes. DCM methylation of C_me_C(A/T)GG penta-nucleotides is a bacterial form of cytosine methylation detected at only very low levels in most mammalian cell types but is maintained when introduced on transgenes in somatic cells without affecting transgene expression^14^. Our study demonstrates that DCM-time machine (DCM-TM) marks both active genes as well as enhancers and confirms that DCM methylation is propagated to daughter cells with limited effect on gene expression. Thus, DCM-TM provides a powerful technology to trace genome-wide gene transcription and enhancer activity back in time without relying on in silico assumptions. We applied DCM-TM to study homeostasis in the small intestine, generating gene and enhancer activity maps that trace the ISC state to the enterocyte state. We found that gene and enhancer activity changes during enterocyte differentiation are not mediated by heterochromatin changes, and we show that the H2A variant H2A.Z is preloaded at ISC enhancers that become activated in the enterocyte. Application of DCM-TM also indicated that commitment of progenitors to the absorptive lineage is a one-way event that does not involve a temporarily dynamic absorptive–secretory intermediate state.

## Results

### DCM–POLR2B labels active genes

**Fig. 1:**
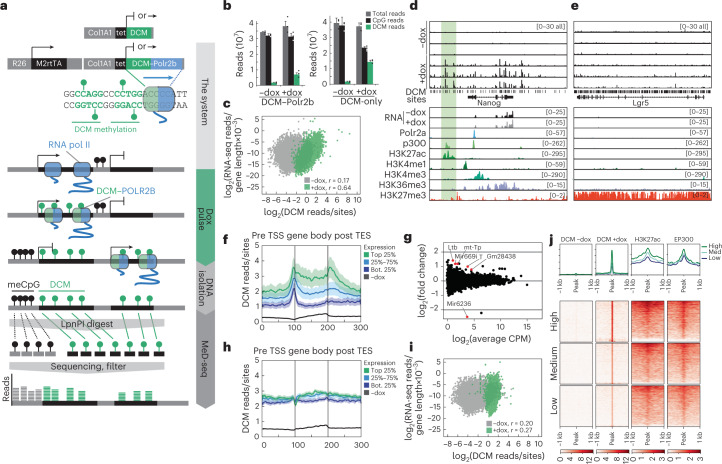
DCM-TM in ESCs. **a**, Overview of the DCM-TM and MeD-seq pipelines. **b**, Induction of DCM labeling measured 5 days after the start of dox treatment in DCM–*Polr2b* and DCM-only transgenic ESC lines (average with s.e.m. plotted, *n* = 3 per condition). **c**, Scatter plot displaying RNA-seq gene expression level in relation to DCM read count per gene before (gray) and after (green) dox induction of DCM–*Polr2b* ESCs. Pearson correlation coefficient is denoted as *r* (**d**,**e**). Genome browser view of DCM-specific MeD-seq reads (*n* = 3), RNA-seq (±dox, average of *n* = 3) and ChIP-seq tracks (ENCODE) in the *Nanog* (**d**, enhancer indicated in green) and *Lgr5* (**e**) loci. **f**, Gene meta-analysis showing distribution of DCM reads of expressed genes split in three clusters based on expression level (top 25%, 25–75% and bottom 25%) in DCM–*Polr2b* ESCs after 5 days of dox treatment (average plotted with ± s.e.m.). **g**, RNA-seq analysis comparing average gene expression values before and 5 days after dox induction (genes indicated in red show significant expression change). **h**, Gene meta-analysis as in **f** for DCM-only ESCs after 5 days of dox treatment (average plotted with ± s.e.m.). **i**, Scatter plot displaying RNA-seq gene expression level in relation to DCM read count per gene before (gray) and after (green) dox induction of DCM-only ESCs. **j**, Heat map showing ChIP-seq overlap (H3K27ac and EP300) with 1-kb regions around enhancer DMRs, which are split in three clusters based on the signal in +dox (second panel). Each profile plot has the same *y*-axis range as its corresponding heat map.

To develop a gene activity tagging system, we fused the bacterial methyltransferase DCM to the N-terminal end of mouse RNA polymerase 2 subunit b (*Polr2b*; Fig. 1a) and introduced this DCM–*Polr2b* fusion gene into the *Col1a1* locus in an embryonic stem cell (ESC) line harboring the m2rtTA *trans*-activator expressed from the *Rosa26* locus (Supplementary Fig. 1a,b)^15^. Addition of doxycycline (dox) leads to expression of the fusion protein at levels lower than endogenous POLR2B, and fusion RNA and protein expression is depleted 24 hours after removal of dox (Supplementary Fig. 1c–e). To detect DCM methylation, we developed methylated DNA sequencing (MeD-seq), a technology based on LpnPI-mediated digestion of CpG and DCM-methylated target sites, resulting in 32 base pair (bp) fragments that are sequenced (Fig. 1a)^16^. LpnPI recognizes 50% of all methylated CpG di-nucleotides (C_me_CG, _me_CGG and G_me_CGC) as well as all DCM-methylated C_me_C(A/T)GG penta-nucleotides. We performed MeD-seq analysis on mouse BAC DNA extracted from a DCM-proficient *Escherichia coli* strain, which revealed a detection efficiency of >99% of all 734 DCM-methylated sequences (Supplementary Fig. 1f), confirming applicability of this technology for DCM methylation detection.

The genome-wide distribution of DCM sites correlates with that of CpG sites, but around gene bodies the distribution of DCM sites is distinctly different from CpG sites (Supplementary Figs. 1i,j and 2). In contrast to the distribution of CpG sites, DCM sites show only a minor enrichment at the transcription start site (TSS). The density of DCM sites shows a linear correlation with gene length, with an average of one DCM site per 512 bp (Supplementary Fig. 1h–j). Addition of dox to DCM–*Polr2b*:m2rtTA ESCs for 5 days resulted in a five-fold induction of DCM methylation genome wide (Fig. 1b, Supplementary Fig. 1g and Supplementary Table 1). In genes with at least ten DCM sites, uninduced gene body DCM methylation displayed little correlation with gene expression, whereas, after dox induction, this correlation became robust (Fig. 1c and Supplementary Fig. 1k). Gene body and genome-wide (2-kb sliding window) DCM labeling was highly reproducible between replicates, and DCM-TM detected nearly all genes expressed in an scRNA-seq dataset from ESCs and could even find additional active genes compared to scRNA-seq and bulk RNA-seq (Supplementary Fig. 1l–n)^17^. DCM methylation was clearly increased in gene bodies of genes expressed in ESCs (*Nanog*, *Zfp42* and *Actb*), whereas no accumulation was observed in genes not expressed in ESCs (*Lgr5* and *Alpi*; Fig. 1d,e and Supplementary Fig. 3a). Gene meta-analysis indicated that the DCM methylation profile before dox induction resembled the distribution of CpG methylation that was present in gene bodies of active genes and possibly introduced as an accidental byproduct of CpG methylation (Fig. 1f and Supplementary Fig. 3c–g)^18^. After induction, the DCM methylation profile displayed increased DCM methylation at the TSSs, gene body and transcription end sites (TESs), with a direct relationship between gene expression and DCM methylation levels (Fig. 1f and Supplementary Fig. 3h). In induced samples, DCM methylation showed an increased correlation with H3K36me3 and RNApol2 chromatin immunoprecipitation followed by sequencing (ChIP-seq) signal, and distribution of DCM methylation appeared as an intermediate between the H3K36me3 and RNApol2 ChIP-seq signal (Supplementary Figs. 3i and 4a).

Only six genes responded with a significant change in gene expression, after induction of the DCM–*Polr2b* fusion gene (Fig. 1g), and DCM methylation did not hamper ESC differentiation into neural progenitor cells (NPCs), showing a highly similar expression change (Supplementary Fig. 3b). To control for an open chromatin effect as reported for DAM-ID^19^, we generated an ESC line with an inducible DCM open reading frame coupled to a nuclear localization sequence (DCM-only) introduced in the *Col1a1* locus of an m2rtTA ESC line (Fig. 1a). In contrast to the DCM–*Polr2b* reporter, activation of the DCM-only construct resulted in an overall increase in DCM methylation and a relative reduction of DCM methylation at promoters and gene bodies (Fig. 1b,h,i and Supplementary Fig. 3j). This reduction was more prominent at promoters of highly expressed genes that might be explained by transcription factor (TF) binding preventing DCM collision and methylation through diffusion of DCM. These results indicate that our DCM–POLR2B fusion protein is efficiently integrated in the RNApol2 complex, labeling active genes with DCM methylation with a minimal effect on gene expression.

### DCM–POLR2B marks active enhancers

Accumulation of DCM labeling was also observed in intergenic regions, and comparison with published ChIP-seq data indicated DCM methylation to accumulate at regions marked by enhancer-specific modifications or protein recruitment (P300, H3K27Ac and H3K4me1; Fig. 1d,j and Supplementary Fig. 4a,b), which is consistent with RNAPol2 recruitment occurring at enhancers^20^. Whole-genome differentially methylated region (DMR) calling between +dox and −dox identified 5,973 regions displaying significantly increased DCM methylation levels, which are enriched for enhancer-specific histone modifications, DNase sensitivity and pluripotency factor binding (Supplementary Fig. 4c). DCM methylation levels were significantly elevated in genes located in closest proximity to these intergenic DCM DMRs, and enhancer density was proportional to activity of the closest gene (Supplementary Fig. 4d,e), indicating that DCM–POLR2B can be applied to trace active enhancers and genes at the same time.

### DCM methylation propagation in vivo

**Fig. 2:**
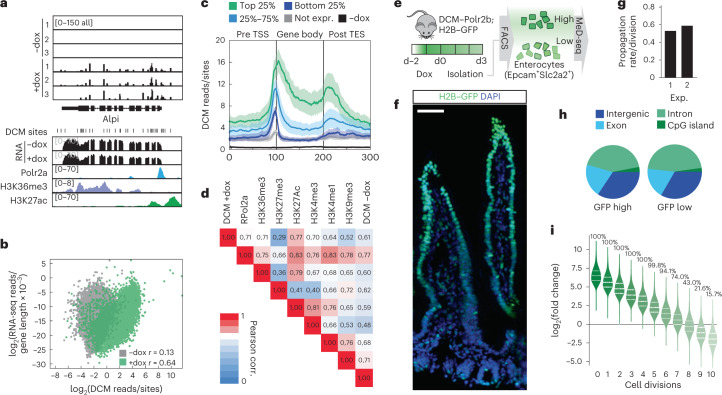
DCM labeling and propagation in the small intestine. **a**, Genome browser view of *Alpi* locus showing DCM-specific MeD-seq reads before and after 1 day of dox treatment (*n* = 3). RNA-seq (±dox, average of *n* = 3), POLR2A, H3K36me3 and H3K27ac ChIP-seq tracks from ENCODE are shown below. **b**, Scatter plot displaying RNA-seq gene expression level in relation to DCM read count per gene before (gray) and after (green) 10 days of dox induction in epithelium of jejunum. **c**, Gene meta-analysis showing distribution of DCM reads in the top 25%, 25–75% and bottom 25% expressed genes after 3 days of dox treatment (average plotted with ± s.e.m.). **d**, Pearson correlation analysis comparing DCM and ChIP-seq read count distribution. **e**, Experimental setup; mice received dox for 2 days with isolation of H2B-GFP_high_ and H2B-GFP_low_ and EPCAM^+^/SLC2A2^+^ cells after a 3-day chase through FACS analysis, followed by DNA isolation and MeD-seq. **f**, Immunocytochemistry detecting H2B-GFP (FITC) and DNA (DAPI) in jejunum of a mouse 3 days after an IP dox pulse (representative image shown from *n* ≥ 5 replicates; scale bar, 50 µm) **g**, Calculated DCM propagation rate in two independent experiments. **h**, Relative distribution of DCM reads in intergenic, exonic, intronic and CpG island sequences in GFP_high_ and GFP_low_ cell fractions. **i**, In silico prediction of DCM labeling levels after each cell division. The fold change between the simulated diluted +dox sample and background levels (−dox) are plotted. The percentage above each violin indicates the percentage of genes that can still be detected based on their estimated fold change.

To monitor accumulation, maintenance and propagation of DCM methylation in vivo in the small intestine, we generated DCM–*Polr2b* transgenic mice. Turnover of the intestinal epithelium is very high; in 5–7 days, the whole epithelium is replaced through differentiation of ISCs into enterocytes and other cell types, requiring 5–6 cell divisions. Total epithelium of jejunum from transgenic mice treated with dox from day 0 through day 18 was isolated through mechanical shearing. MeD-seq analysis indicated DCM methylation to plateau around day 6 with a >25-fold induction over endogenous DCM methylation levels (Supplementary Table 1 and Supplementary Fig. 5a). Similarly as observed in ESCs, DCM methylation of TSS, gene body and TES correlated with gene expression level, POLR2A binding, H3K36me3 deposition and H3K27Ac-enriched regions (Fig. 2a–d). DCM gene density distribution was distinct from CpG methylation, and DCM labeling efficiency of intragenic DCM sites increased to 90% in highly expressed genes (Supplementary Fig. 5b,c). Comparison of RNA-seq data obtained from intestinal epithelium from day 5-induced wild-type, m2rtTA-only and DCM–*Polr2b*:m2rtTA mice revealed a relatively small group of 148 genes showing differential gene expression related to the induction of the DCM–*Polr2b* fusion gene, of which a part could be related to the effect of dox (Supplementary Fig. 5d).

To determine the in vivo DCM methylation propagation rate, we generated compound m2rtTA;H2B-GFP;DCM-*Polr2b* transgenic reporter mice with dox-inducible H2B–GFP and DCM–*Polr2b* alleles. This enabled simultaneous tracking of cell division and DCM methylation profiles in a pulse-chase experiment. The DCM propagation rate of cells in the intestinal epithelium could be established by determination of the relative loss of DCM labeling (MeD-seq detects only fully methylated CCA/TGG sites) in relation to the H2B–GFP signal loss in enterocytes (Fig. 2e). To isolate enterocytes, sequential purification of the small intestinal epithelium was performed, followed by fluorescence-activated cell sorting (FACS) of EPCAM^+^/SLC2A2^+^ (GLUT2) enterocytes (Supplementary Fig. 5e–g,k). Comparison of RNA-seq data of isolated cells with published scRNA-seq data obtained from intestinal epithelium confirmed proper isolation of proximal enterocytes (Supplementary Fig. 5e)^21^. Two pulse-chase experiments were performed by 48 hours of dox administration, followed by a 3-day chase and isolation of GFP_high_ and GFP_low_ populations of EPCAM^+^/SLC2A2^+^ (GLUT2) enterocytes for MeD-seq analysis (Fig. 2e,f). This analysis revealed an average DCM methylation propagation rate of 56% per cell division (Fig. 2g and Supplementary Fig. 5h–j). We found no difference in the propagation rate for specific genomic regions, such as gene bodies, exons, introns, CpG islands or intergenic regions (Fig. 2h). In silico dilution experiments indicated that, with a 56% propagation rate, 74% of the active genes can still be detected after seven cell divisions, indicating that our method is compatible with cell state tracing across temporal windows of multiple cell divisions (Fig. 2i).

### Gene activity dynamics in ISC-to-enterocyte differentiation

**Fig. 3:**
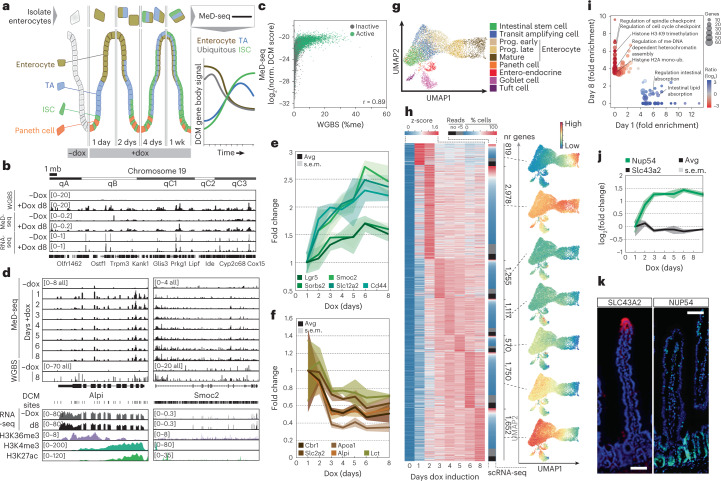
DCM–Polr2b labeling reveals gene activity maps from ISC to enterocyte. **a**, Overview of experimental procedure; mice are labeled with dox and sacrificed at different timepoints to isolate EPCAM^+^/SLC2A2^+^ enterocytes that are subjected to MeD-seq. ISC, TA, enterocyte and ubiquitously expressed genes are expected to display different dynamic behavior in time. **b**, Genome browser view of chromosome 19 showing WGBS (*n* = 1), MeD-seq (average of *n* = 3) and RNA-seq (average of *n* = 3) read count distribution on untreated and 8-day dox-treated enterocytes. **c**, Correlation plot of DCM gene body labeling comparing MeD-seq and WGBS. Genes that are significantly detected using MeD-seq are highlighted in green. **d**, Genome browser view of the average normalized MeD-seq DCM (average of *n* = 3), WGBS (*n* = 1), RNA-seq (average of *n* = 3) and ChIP-seq (H3K36me3, H3K4me3 and H3K27ac) reads in the *Smoc2* and *Alpi* loci at different timepoints after the start of dox treatment. **e**,**f**, DCM labeling (fold change in DCM reads relative to total and normalized to *t* = 1 day) of ISCs (**e**) and enterocyte-specific genes (**f**). **g**, UMAP of jejunum scRNA-seq data showing clusters annotated as specific cell types. **h**, DCM labeling of all significantly labeled genes (negative (*t* = 0) samples compared to all days after the start of dox treatment) clustered according to the maximum DCM signal. For each cluster, the capture by scRNA-seq is shown as the number of genes without reads (black), expressed in five cells with fewer than five reads (gray) and, when expressed, the percentage of cells with signal (blue to red), and average expression of clustered genes is plotted in the UMAP shown in **g**. **i**, Top 200 normalized fold-enriched GO terms for each set of genes peaking at day 1 and day 8. Circle size represents gene number per GO term, and color bar displays the ratio of gene count between day 1 and day 8. **j**,**k**, DCM labeling (**j**) and validation by immunocytochemistry (**k**) of SLC43A2 and NUP54 expression (FITC; DNA is DAPI stained; representative image shown from *n* ≥ 5 (NUP54) or *n* = 3 (SLC43A2) replicates; scale bars, 50 μm).

We tested the DCM-TM technology after differentiation of ISCs into enterocytes that are eventually shed from the top of the villi. A continuous dox pulse experiment was performed in triplicate with isolation of enterocytes at different timepoints across an 8-day window (Supplementary Table 1 and Fig. 3a). In this setting, turnover of the fusion protein is not required, and non-dividing cells do not affect the assay. MeD-seq was performed on DNA isolated from enterocytes, followed by normalization for DCM induction efficiency (Supplementary Fig. 6a and Supplementary Table 2). At day 8 of induction, we observed a 24-fold induction of DCM methylation in isolated enterocytes. Comparison of our MeD-seq results with whole-genome bisulfite sequencing (WGBS) on DNA isolated from uninduced and day 8-induced enterocytes indicated a very high correlation between the MeD-seq and WGBS technologies (Pearson *r* = 0.89; Fig. 3b–d and Supplementary Fig. 6b,c). We found an average labeling efficiency of DCM sites in active genes of 8.7%. Linkage analysis of the methylation status of neighboring DCM sites in genic WGBS reads revealed a preference for *cis*-DCM methylation but also indicated DCM–POLR2B labeling efficiency to be below 100%, likely explaining the correlation found between DCM labeling and gene expression level (Supplementary Fig. 6d). Comparison of DCM methylation capture between MeD-seq and WGBS showed that, with a similar number of sequenced reads, MeD-seq is capable of detecting at least 120 times more DCM methylation labels, demonstrating its cost-effective value (Supplementary Fig. 6a,b).

In a continuous dox pulse experiment, DCM methylation levels of stem-cell-specific genes are expected to increase relative to the total pool of MeD-seq reads as their profile will be propagated in the transit-amplifying (TA) cells and enterocytes (Fig. 3a). As a consequence, MeD-seq reads from enterocyte-specific genes as well as ubiquitously expressed genes are expected to decline relative to the total pool of MeD-seq reads obtained from FACS-sorted enterocytes. Indeed, stem cell markers *Lgr5*, *Sorbs2*, *Smoc2* and *Slc12a2*; WNT target gene *Cd44*; and ephrin receptors *EphB2* and *EphB3* displayed an increase in DCM gene body labeling, reaching a maximum signal at day 6 (Fig. 3d,e and Supplementary Fig. 6e). Ephrin receptor ligands expressed in the villus, *Efnb1* and *Efnb2* and enterocyte markers *Cbr1*, *Slc2a2*, *Apoa1*, *Alpi* and *Lct* displayed a decrease in DCM methylation (Fig. 3d,f and Supplementary Fig. 6e). Ubiquitously expressed genes resembled enterocyte-specific genes but with slower kinetics and less dynamic behavior (Supplementary Fig. 6f). Finally, genes associated with other differentiated cell types or cell types implicated in injury-induced plasticity, including the Paneth, goblet, tuft, enteroendocrine and +4 cell, either did not increase over time or remained below background levels, indicating no role for these cell types in intestinal homeostasis in the measured timespan (Supplementary Fig. 6g,h). One clear exception was *Bmi1*, contrasting with other +4 cell markers in behavior resembling other ISC genes. This emphasizes a role for *Bmi1* in ISC homeostasis in line with studies, indicating *Bmi1* to be essential for ISC maintenance and intestinal homeostasis^22^.

To generate temporal gene activity maps throughout ISC-to-enterocyte differentiation, genes with a DCM signal significantly higher than background levels were clustered according to their temporal signal strength based on their maximum signal day. The average expression of the different gene clusters was then displayed on a uniform manifold approximation and projection (UMAP) based on scRNA-seq data from intestinal epithelium as normalized read count distribution per cell type (Fig. 3g,h)^21^. This analysis showed that genes with a temporal methylation profile peaking at day 1 (cluster 1) are enriched in enterocytes, whereas genes that peak at day 6 (cluster 6) and more prominently at day 8 (cluster 8) are enriched in ISCs (Fig. 3h), suggesting that our analysis traces all the way back through intestinal development. Genes with a maximum temporal signal at day 2 displayed the highest expression level and were expressed in most single cells across different cell types, indicating that this cluster mostly represents ubiquitously expressed genes (Fig. 3h and Supplementary Fig. 6i). DCM-TM detects more genes than detected by scRNA-seq, which misses lowly expressed genes due to limited sensitivity (Fig. 3h)^17^. Gene Ontology (GO) analysis revealed enrichment of gene sets for cell cycle and heterochromatin associated with the ISC at late timepoints (cluster 8), and gene sets for the digestive system showed clear enrichment at early timepoints (cluster 1; Fig. 3i and Supplementary Table 5). Using immunocytochemistry, we confirmed exclusive expression of cluster 1-specific proteins (SGLT1 and SLC43A2) in the villus and cluster 6 and 8 proteins (NUP54 and GNL3) in the crypt (Fig. 3j,k and Supplementary Fig. 6j–l). We conclude that DCM-TM is capable of tracing gene activity back over multiple cell divisions from the enterocyte to the ISC.

### Enhancer activity dynamics in ISC differentiation

**Fig. 4:**
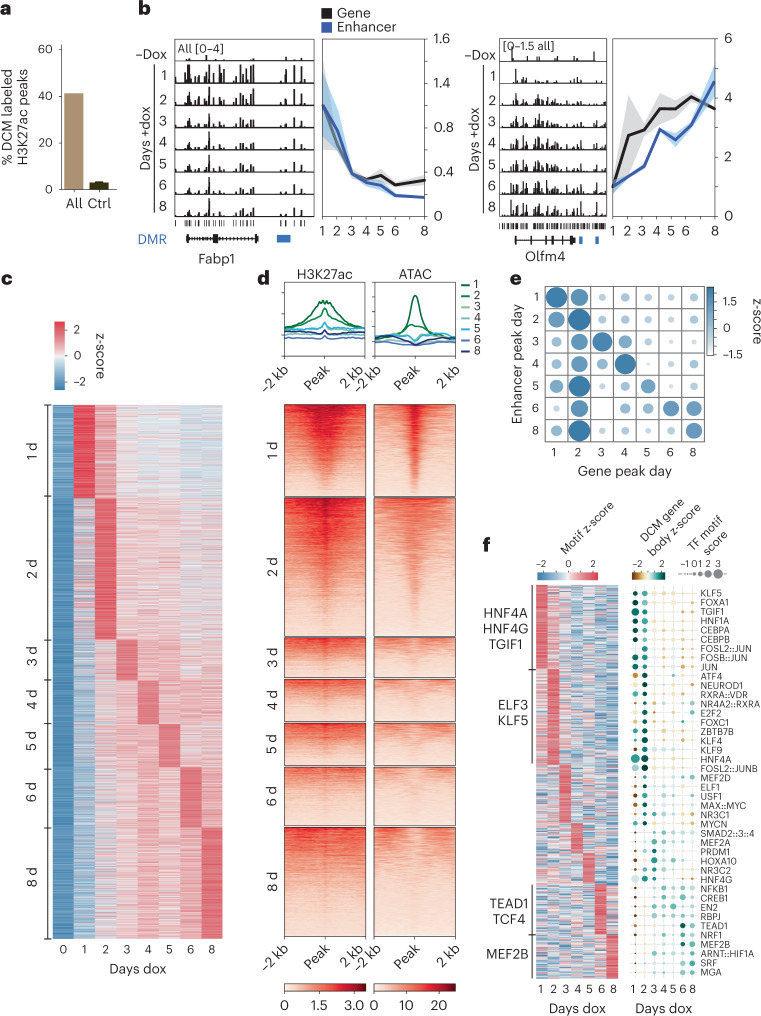
Temporal changes in TF and enhancer activity from ISC to enterocyte. **a**, Percentage of H3K27ac peaks that are labeled by DCM (that is, peaks with ≥1 significant DCM site <750 bp from peak). Random control based on 100 sets of reshuffled H3K27ac peaks is added to show expected random overlap (*n* = 1 calculation for all, *n* = 100 calculations on randomized data as control, mean ± s.d.). **b**, Left panels display genome browser view showing DCM labeling of enterocyte-specific (*Fabp1*) and ISC-specific (*Olfm4*) genes with nearby enhancers (marked in blue) showing coordinated behavior in time (average of *n* = 3). Right panels show the profiles over time for each gene and of the closest significantly labeled DCM sites (average plotted with ± s.e.m.). **c**, Heat map of DCM labeling of enhancers (z-scores of mean-normalized DCM reads). **d**, Heat map showing H3K27ac ChIP-seq and ATAC-seq overlap with the regions around enhancer DMRs peaking at different days of dox induction. Each profile plot has the same *y*-axis range as its corresponding heat map. **e**, Correlation between gene peak day and peak day of closeby enhancers (z-score of proportion of enhancers per day). **f**, Heat map showing TF motif dynamics observed in intergenic DCM DMRs in time (left) and combined analysis of motif enrichment and DCM gene body labeling dynamics of TFs displaying a positive correlation in time (right).

We next explored whether enhancer activity could be tracked across ISC-to-enterocyte differentiation using ChIP-seq and assay for transposase-accessible chromatin using sequencing (ATAC-seq) data generated in epithelium isolated from the villus^23^. We found a clear correlation among H3K27ac, which marks active promoters and enhancers^24^, DNA accessibility and DCM methylation (Supplementary Fig. 7a,d)^23^. Forty-two percent of the H3K27ac peaks were enriched for DCM (with 80% of the high-DCM cluster labeled), whereas most of the remaining enhancer peaks lacked sufficient DCM sites for high-confidence analysis of their state (Fig. 4a and Supplementary Fig. 7b–d). Interestingly, DCM-TM detected a limited number of bivalent enhancers marked by both H3K27ac and H3K9me3, suggesting that POLR2B is, in rare cases, recruited to poised enhancers. DCM methylation was evident at known enhancers near enterocyte (*Fabp1* and *Cbr*), ubiquitous (*Actb*) and ISC (*Olfm4* and *Znhit3*) genes, peaking at different timepoints after the start of dox treatment (Fig. 4b, Supplementary Fig. 8a and Supplementary Table 3)^25^. We identified 51,779 intergenic DCM DMRs (>1 kb from TSS) between −dox and +dox (all stages). Clustering of these intergenic DMRs based on their temporal peak values highlighted the dynamic behavior of enhancer activity during cell state specification (Fig. 4c). As expected, enrichment of H3K27ac at DMRs was more pronounced at early than later timepoints, as these DMRs reflect enterocyte-specific and ubiquitous enhancers that are active in villi (Fig. 4d). Interestingly, ATAC-seq analysis indicated that enterocyte enhancers are accessible, whereas enhancers active at earlier stages of differentiation lose accessibility in enterocytes. We did not observe this dynamic behavior in accessibility for TSSs (Supplementary Fig. 8b). Density analysis of enhancers of the different clusters around the different gene clusters showed a coordination in peak days of enhancers and nearby genes in the enterocyte differentiation process (Fig. 4e and Supplementary Fig. 8c). Cluster 2 genes display different enhancer kinetics, as this cluster consists of both ubiquitously expressed and stage-specific genes (Supplementary Fig. 8d). ChromVAR motif analysis on enhancer regions confirmed enrichment of motifs for ISC-specific TFs peaking at day 8 (TCF4 and TEAD1). Enterocyte-differentiation-associated TFs, including ELF3, KLF5 as well as HNF1A/G, known to play a crucial role in enterocyte differentiation, peak at early timepoints (Fig. 4f, Supplementary Fig. 8e and Supplementary Table 4)^26,27^. In addition, by selecting TFs displaying coordinated timing of gene body and enhancer DCM labeling (Pearson *r* > 0.3), we were able to identify *Mef2b* and *Tgif1* as potentially novel candidate TFs in ISC homeostasis and enterocyte differentiation, similar to their proposed roles in other systems (Fig. 4f)^28,29^. In addition, reverse-coordinated peak timing (Pearson *r* < −0.3) with maximum DCM gene body labeling in ISCs and maximum motif labeling in enterocytes identified known and putative new repressors (*Atf7*, *Glis2* and *Mixl1*) of the ISC state^30^(Supplementary Fig. 8f). These results show that DCM-TM can be used to detect enhancer activity and relate these to underlying TF dynamics.

### Chromatin priming of future active enhancers

**Fig. 5:**
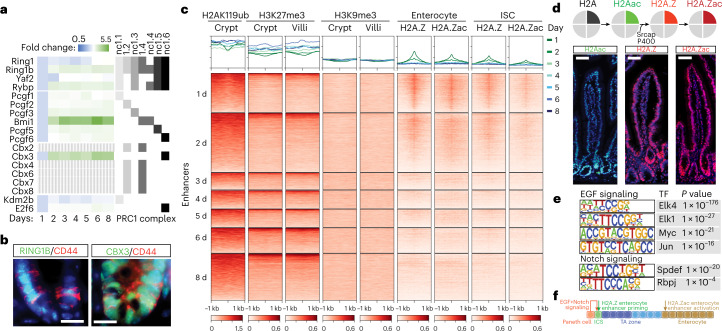
H2A.Z is recruited to enterocyte-specific enhancers in ISCs. **a**, Temporal behavior of DCM methylation (normalized to *t* = 1 day) of members of different PRC1 complexes (genes indicated in dashed gray do not accumulate DCM signal above background). **b**, Immunofluorescence detection of RING1B (FITC), CBX3 (FITC) and CD44 (Texas Red) in the intestinal crypt (DNA = DAPI; representative image shown from *n* ≥ 5 replicates; scale bars, 16 μm). **c**, Heat map showing H2A119ub, H3K27me3 and H3K9me3 ChIP-seq and H2A.Z and H2A.Zac CUT&RUN overlap in indicated cell types with the regions around enhancer DMRs peaking at different days of dox induction. Enhancers were ordered according to H3K27ac and ATAC-seq enrichment (Fig. 4d). Each profile plot has the same *y*-axis range as its corresponding heat map. **d**, Top diagram shows sequential changes from H2A modifications and variant replacement. Bottom panels show immunocytochemistry detecting H2Aac (FITC), H2A.Z and H2A.Zac (both Texas Red) (DNA = DAPI; representative image shown from *n* ≥5 replicates; scale bars, 50 μm). **e**, HOMER motif analysis on H2A.Z enhancer peaks present in enterocytes revealing motif enrichment for TFs downstream of EGF and Notch signaling (*P* values calculated using a hypergeometric distribution). **f**, Model depicting timing of H2A.Z incorporation and acetylation of enterocyte-specific enhancers.

GO analysis of cluster 8 genes indicated enrichment of heterochromatin-associated terms, and gene expression changes in ISC differentiation might, therefore, be directed by dynamic changes in the heterochromatin landscape (Fig. 3i). Polycomb repressive complex 1 and 2 (PRC1 and PRC2) play a crucial role in establishment and maintenance of facultative heterochromatin, and loss of PRC1 members *Bmi1* or *Ring1b* results in ectopic expression of non-lineage genes and loss of the ISC fate^22,31^. DCM-TM indicated *Bmi1* and other members of non-canonical PRC1 (ncPRC1) to show maximum gene body DCM labeling on days 6 and 8, indicative for ISC-specific expression of these factors, and enrichment of ncPRC1 members RING1B and CBX3 was confirmed in CD44^+^ ISCs (Fig. 5a,b and Supplementary Fig. 9a)^32^. Similarly, several HP1-associated factors involved in maintenance of constitutive heterochromatin also belonged to clusters 6 and 8 (Supplementary Fig. 9b) with moderate enrichment of its target H3K9me3 (and no enrichment of H3K9me2) detected in the crypt (Supplementary Fig. 9c). Interestingly, analysis of published ChIP-seq data examining enrichment of H2A119ub and H3K27me3, catalyzed by PRC1 and PRC2, and H3K9me3 specific for constitutive heterochromatin, revealed a lack of dynamic changes in enrichment of all tested modifications at enhancers and promoters at any stage of ISC differentiation (Fig. 5c and Supplementary Fig. 9d–f). These results support a role for ncPRC1 in ISC maintenance by preventing expression of non-lineage genes and suggest a less prominent role for heterochromatin-associated mechanisms in gene regulation directing ISC differentiation^31,33^.

The limited role for heterochromatin-mediated regulation of intestinal enhancers and promoters suggests that activation signals and TF networks may play a more dominant role during ISC differentiation. Histone variant H2A.Z has been implicated in lineage-specific gene activation, where H2A.Z acetylation (H2A.Zac) is associated with enhancer activation^34^. H2A.Z incorporation is mediated by SRCAP and P400 (genes both peaking on days 6 and 8) and preceded by H2A acetylation, which showed marked enrichment in the crypt, whereas HA2.Z and H2A.Zac were more uniformly distributed in the crypts and villi (Fig. 5d and Supplementary Fig. 9g,h). CUT&Tag analysis indicated H2A.Z and H2A.Zac accumulation at ISC and enterocyte-specific TSSs (Supplementary Fig. 9d)^35^. In contrast, H2A.Z preferentially accumulated on enterocyte-specific enhancers (peaking on day 1 or day 2) both in ISCs and enterocytes, with more pronounced H2A.Zac enrichment in enterocytes (Fig. 5c). We found no difference in H2A.Z and H2A.Zac enrichment between enhancers <5 kb or >5 kb away from promoters, as a proxy for H2A.Z1 or H2A.Z2 isoform accumulation^36^. This highlights a distinct role for H2A.Z in enhancer and gene activity regulation and indicates that H2A.Z pre-marks enterocyte-specific enhancers in ISCs, which are activated during ISC differentiation through acetylation. HOMER motif enrichment analysis of H2A.Z enhancer peaks present in enterocytes revealed enrichment of TF binding sites for factors involved in Notch signaling (RBPJ and SPDEF) and several targets of the EGF signal transduction pathway, including ELK1, ELK4, cMYC and cJUN, suggesting a role for these pathways in H2A.Z recruitment and maintenance (Fig. 5e,f). These findings emphasize the presence of an ISC-specific chromatin landscape to maintain ISC stemness and lineage identity and to prepare and delineate enterocyte-specific enhancers and genes for future activation upon cell differentiation.

### The absorptive–secretory switch in ISC differentiation

**Fig. 6:**
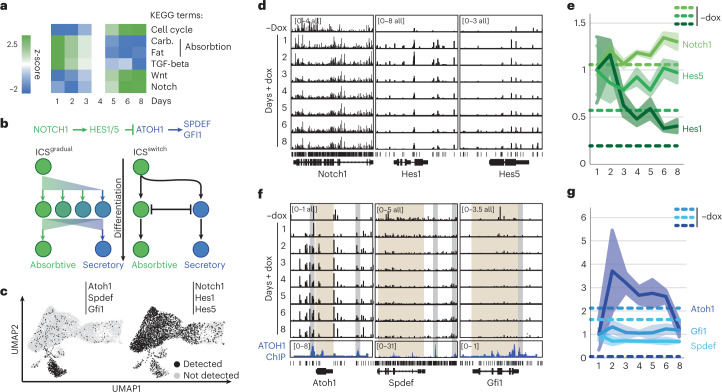
Notch signaling in absorptive versus secretory cell fate decision. **a**, KEGG pathway enrichment analysis at different timepoints after the start of dox treatment. **b**, Notch signaling pathway in ISC differentiation toward absorptive and secretory lineage; two possible mechanisms involving a gradual change or an on/off switch in Notch signaling are shown. **c**, Cells expressing at least one of following three genes—*Atoh1*, *Spdef* and *Gfi1* (left) or *Notch1*, *Hes1* and *Hes5* (right)—in scRNA-seq are shown in UMAP of Fig. 4g. **d**,**e**, Genome browser view of MeD-seq DCM reads (average of *n* = 3) in *Notch1* and its target genes *Hes1* and *Hes5* and quantification of DCM signal normalized to day 1 (dashed lines represent −dox signal per gene, average plotted with ± s.e.m.). **f**,**g**, As in **e**,**f** but now for *Atoh1*, *Spdef* and *Gfi1* (average plotted with ± s.e.m.). Bottom tracks in **f** show ATOH1 ChIP-seq signal from *Atoh1* GFP^+^ cells from the small intestine (ATOH1-targeted regulatory regions are indicated in gray and gene bodies in brown).

To better understand cell state changes in enterocyte differentiation, we performed KEGG pathway analysis on DCM-labeled genes and found enrichment of pathways, including absorption and TGF-β signaling, at early timepoints. Pathways including cell cycle, Wnt, EGF and Notch signaling showed enrichment at later timepoints, consistent with their lineage history (Fig. 6a and Supplementary Table 6). Notch signaling controls the absorptive versus secretory cell fate decision, dictating repression of *Atoh1* in the ISC and enterocyte progenitors through action of *Hes1*, *Hes3* and *Hes5* (Fig. 6b). Loss of contact of proliferating ISCs (Notch^+^) with Paneth cells expressing the Notch ligand *Dll1* leads to downregulation of *Notch1* and subsequent upregulation of *Atoh1* in future secretory cells^8^.

Notch-mediated repression of *Atoh1* and its key target genes *Spdef* and *Gfi1* could involve a gradual transition from a bi-potential progenitor to one cell state or may involve a binary switch toward the absorptive or secretory lineage. scRNA-seq data demonstrated predominant expression of *Atoh1*, *Spdef* and *Gfi1* in secretory cell types but also revealed several cells that appear committed to the absorptive lineage to express at least one of these genes (Fig. 6c). In addition, a few cells express both *Notch1* and *Atoh1* or do not express *Notch1* and *Atoh1* at all, making it difficult to discern how the absorptive–secretory switch is mediated based on RNA velocity or pseudotime analyses (Supplementary Fig. 10a–c)^21,37^. Examination of DCM-TM data indicated that *Notch1* as well as Notch target genes, including *Hes1, Hes3* and *Hes5*, are expressed throughout enterocyte differentiation (Fig. 6d,e). In contrast, DCM methylation in gene bodies of *Spdef* and *Gfi1* never got above background levels (Mann–Whitney significance test, *P* > 0.05), suggesting that their activation results in irreversible commitment toward the secretory lineage. Interestingly, the UMAP representation of DCM-TM also indicated that several genes normally expressed in secretory cell types are transiently activated in enterocyte progenitors, highlighting the bi-potential nature of these progenitors (Fig. 3h and Supplementary Fig. 10d–e). However, our results also indicate that the switch toward the secretory state represents a one-directional switch orchestrated by key regulatory TFs that are never activated in bi-potential progenitors that become enterocytes (Fig. 6f,g).

## Discussion

To facilitate whole-genome cell state tracing, we developed a system to tag transcribed genes with DCM methylation labels to be examined at later stages of development or differentiation in vivo. We applied this DCM-TM technology to perform whole-transcriptome and enhancer activity lineage tracing of specific cell types and demonstrated the possibility to establish TF and signal transduction roadmaps through isolation of a differentiated cell type without the need to isolate progenitor or stem cells and without the need to infer connectivity in silico. We identified novel marker genes for different cell states, and we provide new insights into the transcriptional dynamics during cellular differentiation in the mouse intestine.

Key parameters for a whole-transcriptome lineage-tracing system are the introduction of a label that is normally absent in mammalian cells, is maintained and propagated upon DNA synthesis and does not interfere with gene expression. Our study showed that DCM methylation approaches these criteria simultaneously. DCM methylation is present only at low levels in wild-type ESCs and intestinal epithelium (2–3%), whereas only a five-fold and a 25-fold induction in ESCs and intestinal epithelium, respectively, are sufficient to reliably identify active genes and enhancers and trace their activity back in time. In contrast to other forms of bacterial methylation, which are not propagated, such as DAM^19^, propagation of DCM methylation is 56% in the intestinal epithelium. This is lower than previously described^14^ but sufficient to detect gene body and enhancer labeling over at least seven cell divisions. Lastly, we found that only a limited number of genes are affected by induction of the fusion protein. This lack of interference with transcription might be related to the fact that gene bodies of active genes already accumulate CpG methylation, thought to repel intragenic initiation of RNA pol2 (ref. ^38^). In addition, as the DCM motif is found much less frequently, it may, thus, have only limited effect on transcription.

In this study, we applied DCM-TM to understand the mechanism directing the absorptive–secretory switch in the intestinal epithelium involving the Notch signaling pathway. Activation of *Notch1* and its downstream *Hes* family targets is mediated by cell–cell contact through direct contact of NOTCH1 and its ligand DLL. Notch signaling is required for maintenance of the ISC where Notch ligands are expressed by Paneth cells but also during ISC differentiation to consolidate the absorptive lineage^39^. In the secretory lineage, NOTCH1 is downregulated, resulting in de-repression of *Atoh1* and its downstream targets *Gfi1* and *Spdef*. Our DCM-TM data demonstrate that several secretory cell type specific genes are transiently activated in enterocyte precursors, but also show that Notch signaling remains active throughout enterocyte differentiation and that *Spdef* and *Gfi1* are never activated. This indicates that, during ISC differentiation, at least part of the transcriptional absorptive and secretory programs is activated, but that the establishment of the full transcriptional program is directed by a committed absorptive–secretory lineage fate decision directed by *Notch1* and *Atoh1*. Nevertheless, *Atoh1* is active in ISCs and cells committed the absorptive lineage, probably as a consequence of fluctuating Notch activity and to allow a quick response when NOTCH1 levels decrease below a specific threshold for locking in the secretory state. This finding explains why *Atoh1* lineage-tracing studies revealed ATOH1^+^ cells to contribute to the ISC pool^40^, but our data also indicate that this *Atoh1* expression level is too low to activate *Spdef* and *Gfi1* and lock in the downstream secretory program.

Our study and studies of others indicate a very limited role for complexes in heterochromatin formation and maintenance in ISC differentiation-associated gene expression dynamics. Previous studies involving loss of PRC1 members *Bmi1* or *Ring1b* suggested that PRC1 is required for ISC maintenance by preventing ectopic expression of non-lineage genes^22,31^. Application of DCM-TM revealed that PRC1-mediated gene repression is likely mediated by non-canonical PRC1 complexes that contain RYBP, which catalyzes H2A ubiquitination independently of PRC2. This is in line with the observation that loss of PRC2 and H3K27me3 does not affect H2A119ub in the intestine^41^. The present study also revealed a static landscape of repressive chromatin modifications H2A119ub, H3K27me3 and H3K9me3 in intestinal homeostasis. Similarly, DNA methylation changes were found to be very limited between different epithelial cell types^25^, suggesting that the main role of facultative and constitutive heterochromatin in ISCs is dictating repression of non-lineage genes. Therefore, activation and repression of enhancers and genes in intestinal homeostasis appears to be regulated by other epigenetic mechanisms and TF networks. We found histone variant H2A.Z to be loaded on enhancers in ISCs that are destined to become activated in enterocytes, suggesting that the ISC dictates and limits enhancer activity in its decedents through H2A.Z recruitment to enhancers. The clear enrichment of motifs of EGF and Notch-regulated TFs at these enhancers make both signaling pathways the likely candidate signal for H2A.Z recruitment.

Several cell state tracing technologies detecting the history of gene expression have been described previously. These include CRISPR spacer-mediated recording of DNA or RNA to monitor complex cellular behavior retrospectively, as well as smFISH-based detection of CRISPR–Cas-mediated targeted disruption of expressed recording elements^42,43^. Unfortunately, all these technologies are restricted by a limited number of genes that can be recorded. The recent developments in scRNA-seq provide alternative means to detect cell states and gene expression changes in relation to developmental trajectories. Temporal changes in abundance of spliced and un-spliced gene products^37^ and pseudotime inference have been applied to predict cell trajectories in silico^44,45^. However, these analyses are limited by the temporal resolution and the number of genes detected and, therefore, sometimes fail to detect the changes in gene expression from one cell state to the next and are more difficult to apply along developmental trajectories. The present DCM-TM technology circumvents these issues, providing a genome-wide picture of gene and enhancer activity at any timepoint during development or differentiation. The DCM-TM transgene can be combined with conventional lineage-tracing technologies to fine-map cell fate decisions, or with scMeD-seq or scWGBS, to discriminate between lineage paths and keep track of network changes. In addition, DCM-TM can be applied to follow embryonic development and tissue regeneration, providing a powerful system to identify temporal maps of TF networks and signal transduction pathways that can be used to improve stem cell expansion and cell differentiation models.

## Methods

### Generation of DCM–Polr2b ESC line

DCM and Polr2b sequences were amplified by polymerase chain reaction (PCR) from cDNA. For DCM, the primers 5′-GCTAGCATGGTCGACCAGGAAAATATATCAGTAACCGATTCAT-3′ and 5′-GCGGCCGCTTAGGGCGCGCCTCGTGAACGTCGGCCATGTTGTGCCTC-3′ were used and for Polr2b 5′-GCTAGCATGGGCGCGCCGCAATATGATGAAGACGACGATGAGATCA-3′ and Polr2b reverse 5′-GCGGCCGCCTAGTCGACTCATTCGTGGTGCGATGCTCATGGACAT-3′. The DCM sequence was (XhoI-AscI) inserted in frame 5′ to the *Polr2b* coding region, eliminating the translational stop and generating the DCM–Polr2b fusion gene. The fusion gene was introduced (XhoI-NotI, blunt) into pgk-ATG-frt (Addgene, 20734). This DCM–Polr2b shuttle vector was targeted to KH2 mouse ESCs harboring a frt homing site and hygromycin resistance gene in the *Col1a1* locus using a pCAGGS-flpE flipase expression cassette^15^. Positive clones were identified by PCR using the following primers: Cola1-flpin: 5′-TGCTCGCACGTACTTCATTC-3′ and 5′-GAAAGACCGCGAAGAGTTTG-3′.

Empty clones were excluded using primers: Cola-flpin-empty: F1: 5′-TGCTCGCACGTACTTCATTC-3′ and R1: 5′-GGGGAACTTCCTGACTAGGG-3′.

ESCs were maintained on mouse embryonic fibroblasts in ES medium (DMEM, 15% FCS, penicillin–streptomycin (Invitrogen), 1% non-essential amino acids (Lonza, BE13-114E), LIF (1,000U ml^−1^), 0.1 mM 2-mercaptoethanol (Gibco, 31350-010)) with daily media changes. Induction was achieved by adding dox (final concentration 2 µg ml^−1^; Sigma-Aldrich, D9891) to medium. ESCs were passaged every 4 days using trypsin/EDTA dissociation.

### ESC pulse-chase experiment

DCM-Rpol2b:m2rtTA transgenic ESCs were induced with dox for 24 hours, followed by a chase and collection of cells at 4 hours, 8 hours, 12 hours and 24 hours after wash, RNA isolation, reverse transcription and quantitative PCR with primers detecting:

DCM-Polr2b: Fwd: 5′-GGT TTC GGA CAC TCA GGC-3′, Rev: 5′- AGT GAT CTC ATC GTC GTC TTC A-3′

Gapdh: Fwd: 5′- TGC CCC CAT GTT TGT GAT G-3′ Rev: 5′- TGT GGT CAT GAG CCC TTC C-3′

Hsp90: Fwd: 5′- CCA CCA CCC TGC TCT GTA CTA-3′, Rev: 5′- CCT CTC CAT GGT GCA CTT CC-3′

### Differentiation of mouse ESCs to NPCs

Mouse ESCs were differentiated toward NPCs according to an established differentiation protocol^46,47^. Before differentiation, DCM–Polr2b ESCs were cultured on mouse embryonic fibroblasts in ES medium and induced with dox for 5 days, with medium refreshed every other day. For differentiation, an adaption of published protocols was used (Conti et al.^46^ and Splinter et al.^47^). In brief, cells were trypsinized and pre-plated for 40 minutes to remove mouse embryonic fibroblasts. In total, 8 × 10^5^ cells were plated on gelatinized 10-cm dishes and grown in N2B27 medium for 7 days. N2B27 medium consists of 1:1 DMEM/F12 (Gibco, 31330-038) and Neurobasal Medium (Gibco, 21103-049) supplemented with NDiff Neuro-2 Medium Supplement (200×; Millipore, SCM012), 0.5× B-27 supplement (50×), serum free (Gibco, 17504-044), l-glutamine (100×) (Gibco, 25030-024), 50 mM 2-mercaptoethanol (Gibco, 31350-010) 1 ml and penicillin–streptomycin. Afterwards, cells were incubated with Accutase cell detachment solution (Millipore, SCR005) at room temperature until detaching and centrifuged for 5 minutes at 188*g*. In total, 3 × 10^6^ cells were resuspended in N2B27 medium + EGF (10 ng ml^−1^; PeproTech, 315-09) and FGF2 (10 ng ml^−1^; PeproTech, 100-18B) and grown in suspension in 10-cm dishes. The cells form aggregates, and, after 3 days, the aggregates were collected and treated for 5 minutes with Accutase at room temperature. Pelleted cells were resuspended in N2B27 medium + EGF/FGF2 and grown on laminin-coated (Sigma-Aldrich, L2020) plates with media change every other day. After 14 days, the NPCs were harvested, and RNA was isolated using the ReliaPrep RNA Cell Miniprep System (Promega, Z6012) using the manufacturerʼs protocol. As a negative control, non-induced DCM–Polr2b ESCs were used.

### Polr2b–DCM transgenic mice

Expression of the DCM–Polr2b fusion transgene in *DCM–Polr2b:m2rtT*A mice was mediated by addition of dox to the drinking water (2.0 mg ml^−1^, 2% sucrose). Mice were sacrificed by cervical dislocation, after which the jejunum was isolated, and 3–4 cm of proximal jejunum was used for either isolation of total epithelium or villi-enriched isolation. Total jejunum samples were used for DNA and/or RNA isolation; villi samples from jejunum were either used for DNA and/or RNA isolation or dissociated into single cells for FACS. Total epithelium was isolated using chelation Ca^2+^ ions to weaken cell adhesions and subsequent mechanical separation as previously described^48^. For villi isolation from jejunum, we continued with an additional protocol to separate villi from crypts and, if needed, followed by single-cell isolation^49^. Unless stated otherwise, all incubation steps were performed at 4 °C and centrifugation for 5 minutes at 200*g* and 4 °C. The jejunum was collected, flushed with cold 1× PBS, opened longitudinally and cut into pieces of approximately 1 cm. The pieces were incubated in 2 mM EDTA/PBS for 30 minutes on a shaker. After washing twice with cold PBS, the fragments were resuspended in 5 ml of PBS, and villi were mechanically separated with a 10-ml serological pipette. Villi were collected and supplemented with Dispase II (0.4 mg ml^−1^, Sigma-Aldrich). After 30-minute incubation on a shaker at 120 cycli per minute at 37 °C, the Dispase II reaction was stopped by addition of FCS (5% final concentration). Cells were filtered through a 40-uM cell strainer, counted and collected in 2% FCS/PBS at 1 × 10^6^ cells per milliliter. Cells were incubated with antibodies (Epcam, Cd326, eFluor 450, eBioscience, from Thermo Fisher Scientific, 48-5791-82) and SLC2A2 (GLUT-2, cy5 from Bioss, bs-0351R-Cy5) for 45 minutes at 4 °C, protected from light and washed twice in 1 ml of cold PBS. After final centrifugation (5 minutes at 200*g* and 4 °C), cells were resuspended in 1 ml of cold PBS and filtered through a 40-uM cell strainer before proceeding with FACS. Cells stained for CD326 and GLUT-2 were FACS sorted using a BD FACSAria II version 9.0.1, using FlowJo 10.7.2, and double-positive cell populations were isolated, collecting >10,000 enterocytes per timepoint. DNA was isolated using a QIAamp DNA Micro Kit (Qiagen, 56304) according to the manufacturer’s protocol. RNA was isolated by a TRIzol extraction. All animal experiments were approved by the Dutch Central Committee on the Ethics of Animal Experiments (AVD10100202115681).

### Western blot analysis

For western blot analysis, total protein of mESC DCM–Polr2b was isolated at different timepoints after dox induction (2 µg ml^−1^; Sigma-Aldrich, D9891) with RIPA buffer (Abcam, ab288006). The total protein was run on NuPAGE 3–8% Tris-Acetate Gel (Invitrogen, EA03755BOX) and transferred to a PVDF membrane overnight at 4 °C and constant current (60 mA). The membrane was blocked for 30 minutes at room temperature in blocking buffer (1.3 g of non-fat dry milk in 50 ml of 1× Tris-buffered saline) and probed with POLR2B (Thermo Fisher Scientific, PA5-30122) or DCM (Cusabio, CSB-PA365131XA01ENV) primary antibodies (1:1,000) in blocking buffer + 0.1% Tween for 2 hours at room temperature. Subsequently, the membrane was incubated with anti-rabbit HRP secondary antibody (Sigma-Aldrich, A6154, 1:5,000) in blocking buffer + 0.1% Tween for 1 hour at room temperature. As an internal protein control, β-actin was used: monoclonal anti-β-actin peroxidase antibody (Sigma-Aldrich, A3854, 1:7,500). Protein was detected with SuperSignal West Femto Maximum Sensitivity Substrate (Thermo Fisher Scientific, 34094) and an Amersham Imager 600 (Amersham Biosciences).

### Immunohistochemistry on cryosections

Mice were sacrificed, and the proximal jejunum was collected and flushed with cold PBS, opened longitudinally and cut into 1-cm pieces. The tissue pieces were fixed for 3 hours at 4 °C in 4% PFA/PBS and subsequently rotated overnight at 4 °C in 4% PFA/30% sucrose/PBS. Fixed tissue pieces were embedded in OCT, and 8-µm-thick slices were sectioned onto silane adhesive slides and fixed with cold methanol for 20 minutes. Sections were washed three times for 5 minutes with PBST (1× PBS, 1% BSA and 0.1% Tween 20) and were incubated for 10 minutes at room temperature in PBSTX (1× PBS, 0.5% Triton X-100 and 1% BSA) for permeabilization. After three PBST washes of 5 minutes, sections were blocked for 1 hour at room temperature in blocking solution (1× PBS, 0.1% Tween 20 and 5% normal goat serum (Sigma-Aldrich, G9023)). This was followed by an overnight incubation at 4 °C with primary antibody in blocking solution. Sections were washed three times for 5 minutes in PBST and incubated for 1 hour at room temperature with secondary antibody (1:500) in blocking solution. Sections were then washed with PBST three times for 5 minutes and covered with ProLongGold antifade reagent with DAPI (Invitrogen, P36931).

### Immunohistochemistry on paraffin sections

Jejunum was collected and prepared as described above. The tissue fragments were fixed overnight at 4 °C in 4% PFA/PBS and embedded in paraffin according to standard histologic protocols. Then, 6-µm sections on silan adhesive slides were incubated for 1 hour at 60 °C, deparaffinized and rehydrated in serial xylene and ethanol steps and washed three times in PBS. Sections were incubated for 15 minutes at room temperature in ProtK (1 µg ml^−1^ in PBS) and washed four times for 2 minutes in dH_2_O. Epitope retrieval was performed in 1× sodium citrate buffer (0.01 M) pH 6 + 0.05% Tween, by using the microwave at 900 W for 20 minutes. The sections were cooled down in the buffer to room temperatuire for 1 hour, washed three times in PBS for 5 minutes and blocked for 30 minutes at room temperature in 10% normal goat serum/5% BSA/PBS (for the GNL3/nucleostemin, antibody donkey serum (Sigma-Aldrich, D9663) was used). This was followed by an overnight primary antibody incubation at 4 °C in 5%BSA/PBS. The sections were washed three times for 5 minutes in PBS, incubated for 1 hour at room temperature with secondary antibody (1:500) in 1% BSA/PBS, washed again three times for 5 minutes in PBS and covered with ProLongGold antifade reagent with DAPI. For the staining of GNL3/nucleostemin in combination with HCAM, a TSA Biotin Systems Kit was used according to the manufacturerʼs protocol.

**Table:** 

Antibody	Source	Dilution
Rabbit anti-SGLT1	Alomone Labs, AGT-031	1:200
Rabbit anti-SLC43A2	MyBioSource, MBS9210948	1:50
Rabbit anti-SLC2A2/Glut2 Cy5	Bioss, bs-0351R-Cy5	1:50, 5 µl per 1 × 10^6^ cells
Rat anti-EpCam 450	Invitrogen, 48-5791-82	1:50, 2 µl per 1 × 10^6^ cells
Rat anti-CD31-BV421	BD Horizon, 563356	0.2 µl per 1 × 10^6^ cells
Rat anti-CD45-BV421	BD Horizon, 563890	0.2 µl per 1 × 10^6^ cells
Rat anti-TER119-BV421	BD Horizon, 563998	0.2 µl per 1 × 10^6^ cells
Rat anti-CD24-APC	BioLegend, 562349	0.4 µl per 1 × 10^6^ cells
Rat anti-CD117-PE (cKit)	BioLegend, 105808	0.3 µl per 1 × 10^6^ cells
Goat anti- GNL3/nucleostemin	R&D Systems, AF1638	1:50
Rabbit anti-Nup54	Novus, NBP1-85899	1:50
Rabbit anti-CBX3	Proteintech, 11650-2AP	1:20
Rat anti-HCAM	Santa Cruz Biotechnology, sc-18849	1:50
Rabbit anti-H2AK119Ac	Gift from Zu-Wen Sun	1:500
Rabbit anti-H3K9me2	Upstate, 07-212	1:100
Rabbit anti-H3K9me3	Diagenode, cs-056-050	1:200
Rabbit anti-histone H2A.Z	Abcam, ab4174	IHC: 1:500, CUT&Tag: 1:100
Rabbit ant-acetyl histone H2A.Z	Merck, ABE1363	IHC: 1:500, CUT&Tag: 1:100
Rabbit anti-H3K27me3	Cell Signaling Technology, 9733	1:100
Rabbit α-mouse antibody	Abcam, ab46540	1:100
Mouse Ring1B clone #3	Atsuta, T., Fujimura, Y., Moriya, H., Vidal, M., Akasaka, T. & Koseki, H. Production of monoclonal antibodies against mammalian Ring1B proteins. *Hybridoma* **20**, 43–46 (2001)	1:2
Goat anti-rat Alexa 488	Invitrogen, A-11006	1:500
Goat anti-rabbit Alexa 488	Invitrogen, A-11008	1:500
Streptavidin Alexa 488	Invitrogen, s-32354	1:200
Goat anti-rat Alexa 546	Invitrogen, A-11081	1:500
Goat anti-rabbit Alexa 546	Invitrogen, A-11010	1:500
Donkey anti-rabbit Alexa 546	Invitrogen, A-10040	1:500
Donkey anti-goat Alexa 555	Invitrogen, A-21432	1:500
Rabbit anti-rat biotinylated	Dako, E0468	1:200
TSA Biotin Systems	PerkinElmer, NEL700A	

### MeD-seq sample preparations

MeD-seq analyses were essentially carried out as previously described^16^. At least 10 ng of DNA was digested by LpnPI (New England Biolabs). Stem-loop adapters were blunt-end ligated to repaired input DNA and amplified to include dual-indexed barcodes using a high-fidelity polymerase to generate an indexed Illumina next-generation sequencing (NGS) library. The amplified product was purified on a Pippin HT system with 3% agarose gel cassettes (Sage Science). Multiplexed samples were sequenced on Illumina HiSeq 2500 systems for single reads of 50 bp according to the manufacturer’s instructions. Dual-indexed samples were demultiplexed using bcl2fastq (Illumina). All experimental timepoints were performed in triplo.

### MeD-seq data analysis

Data processing was carried out using custom scripts in Python and MATLAB. Raw FASTQ files were subjected to Illumina adaptor trimming, and reads were filtered based on LpnPI restriction site occurrence between 13 bp and 17 bp from either 5′ or 3′ end of the read. DCM methylation data (CCWGG sites) and CpG methylation data (CCG, CGG and GCGC sites) were separated during filtering and mapped separately to mm10 using bowtie2 (ref. ^50^). Genome-wide individual DCM site scores were used to generate read count scores for all annotated genes from UCSC (GRCm38.p2). BAM files were generated using SAMtools version 0.1.19 for visualization in IGV^51,52^. Because DCM and CpG methylation can be detected separately using MeD-seq, DCM enrichment was determined by either data normalization using CpG read coverage (for absolute DCM enrichment) or DCM read coverage (for relative DCM enrichment) between samples. For both situations, normalization is done using reads per million (RPM), where absolute DCM levels indicate the level of DCM–Rpol2b induction, and relative DCM levels are used to correct for differences in DCM–Rpol2b induction between mice and/or timepoints.

For gene meta studies, intragenic distribution of DCM (or CpG) reads was shown by generating 100 bins of 100 bp (10 kb) either upstream of the TSS or downstream of the TES. Gene body bins were generated using genes with a minimal gene size of 100 bp and dividing each gene body into 100 bins of 1% of the total gene body size; genes with overlapping gene bodies were excluded. For each bin, the number of DCM (or CpG) reads was plotted after adjusting for the DCM (or CpG) site frequency per bin. To compare pre-TSS and post-TES regions (10 kb) to the gene body regions, DCM site count for each gene body bin is adjusted for gene size and the 10-kb region. Subgroups were based on RNA expression data of the corresponding gene. Distribution of DCM reads across peaks from ChIP-seq data were generated accordingly, using genome-wide ChIP-seq peak boundaries instead of annotated genes. All ChIP-seq datasets were downloaded from the ENCODE portal^53^ (https://www.encodeproject.org; mouse ESC: ENCSR000CCC, ENCSR000CCD, ENCSR000CGN, ENCSR000CGO, ENCSR000CFZ, ENCSR000CGQ, ENCSR000CFN and ENCSR000CGR; mouse intestine: ENCSR159RVN, ENCSR198ACZ, ENCSR311VKI, ENCSR642VYW, ENCSR389EYR, ENCSR483KOD and ENCSR000CEE; ATAC-seq: ENCSR079GOY; and Lgr5^+^ ATAC-seq from the Gene Expression Omnibus (GEO): GSE83394.) For DCM methylation, ChIP-seq data comparing DCM and ChIP-seq read counts from the ChIP-seq peaks were used. The log_10_ of (DCM read counts in peak / DCM sites in peak) was plotted with the log_10_ of (ChIP-seq read counts in peak / peak length), followed by a Pearson correlation coefficient calculation (removing outliers with z-score >4).

To visualize relative DCM methylation changes over time during dox induction, we used triplicates of DCM read counts per gene. DCM read counts per gene were normalized for the total amount of DCM read counts per timepoint; mean DCM methylation levels were calculated; and the s.e.m. was used as measure for variability. Fold changes in mean DCM methylation per timepoint were calculated versus day 1, which was set as calibrator. DCM genes were selected for further analysis when the *P* value of Mann–Whitney *U*-test was below 0.05, using the negative DCM days as set X (*n* = 3) and all other DCM days as set Y (*n* = 21). MeD-seq sequence data are deposited at the National Center for Biotechnology Information (NCBI) with GEO accession number PRJNA615329.

### DCM propagation rate in the small intestine

Pulse-chase experiments were performed with m2rtTA;H2B-GFP;DCMPolr2b compound transgenic reporter mice through an intraperitoneal (IP) dox injection. Enterocytes were isolated by FACS of Epcam^+^/SLC2A2^+^ (GLUT2) cells 3 days after a dox IP injection. The ratios of the GFP_high_ and GFP_low_ populations of sorted fractions were established, and DNA was isolated for MeD-seq analysis. The DCM methylation propagation rate was then calculated based on the DCM and CpG methylation read count ratio in relation to the number of cell divisions according to the following equations: $${{{\mathrm{division}}}}\;{{{\mathrm{nr}}}} = {{{\mathrm{log}}}}0.5\left[ {\begin{array}{*{20}{c}} {{{{\mathrm{GFPlow}}}}} \\ {{{{\mathrm{GFPhigh}}}}} \end{array}} \right]$$

$${{{\mathrm{propagation}}}}\;{{{\mathrm{rate}}}} = \root {{{{{\mathrm{division}}}}\;{{{\mathrm{nr}}}}}} \of {{\left[ {\begin{array}{*{20}{c}} {{{{\mathrm{DCMlow}}}}} \\ {{{{\mathrm{DCMhigh}}}}} \end{array}} \right]}}$$

We simulated what the DCM labeling levels would be after each cell division based on an average propagation rate of 56% to discover how many active genes could still be detected. Random subsets of the 2-day dox-induced samples (*n* = 3) were taken using bbtools version 37.62 reformat.sh using a ‘samplerate’ of 0.56^division nr^. From each simulated dataset, the number of reads overlapping each gene was counted using BEDTools version 2.29.2 intersect, and the read counts were normalized for the sequencing depth using the number of CpG reads of the original sample^54,55^. Finally, the fold change between the simulated subsets (*n* = 3) and the non-induced samples (*n* = 3) was plotted for all genes active in the complete MeD-seq dataset and peaking on day 2. For this list of genes, we calculated which percentage of genes had a fold change above 1, indicating that the simulated induced samples still have higher DCM methylation levels compared to the non-induced samples, and labels can still be detected.

### RNA-seq analysis

Total RNA (1,000 ng per sample) was extracted in triplicate for the ESCs, NPCs and transgenic mouse samples. After rRNA depletion, sequencing libraries were prepared using the KAPA RNA Hyper Prep Kit with RiboErase. Sequencing was performed according to the Illumina TruSeq Rapid version 2 protocol on the HiSeq 2500 with a single-read 51-bp and 7-bp index.

Low-quality reads and contaminants (including sequence adapters) were removed using Trimmomatic. On average, 20 million reads per sample passed the quality assessment and were aligned to the mm10 genome using hisat2 version 2.1.0 (ref. ^56^). Transcript abundance level (transcript count) was generated using HTSeq version 0.9.1 (ref. ^57^). The transcript counts were further processed using the R software environment for statistical computing and graphics (version 3.4.0). Data normalization was performed using an EDASeq R package, and differential expression analysis was performed using an EdgeR R package^58^, using the negative binomial general linear model (GLM) approach. Differentially expressed genes with false discovery rate (FDR) ≤ 0.05 (Benjamini–Hochberg multiple testing correction, expression level in control samples >1 counts per million (CPM)) were retained and used for further processing, GO and pathway analysis. RNA sequence data are deposited at the NCBI with GEO accession number PRJNA615329.

### scRNA-seq analysis

For validation and visualization of the DCM profiles, we downloaded scRNA-seq data from Haber et al.^21^ (GSE92332). Visualization of the cells was done using Monocle3 version 0.2.0 (ref. ^59^) and UMAP version 0.1.4 (ref. ^60^). We first pre-processed the scRNA-seq count matrix using a principal component analysis (PCA) with 75 dimensions and corrected for biases using batch as alignment group and the number of genes per cell as residual model formula_str. UMAP was run on this pre-processed matrix with the following settings: min distance of 0.8, n_neighbors of 120 and the cosine metric. The first two UMAP components were plotted using the clustering labels from Haber et al. as cell labels, which were merged when annotated clusters contain similar cell types.

The correlation between the RNA-seq data and the scRNA-seq data was plotted using custom Python scripts. For all genes with at least ten reads across all cells in the scRNA-seq dataset, we calculated the average TPM across replicates using the RNA-seq data. For each cell, the Pearson correlation between the scRNA-seq counts and the average TPM values from the RNA-seq was calculated. The Pearson correlation per cell was then visualized on the UMAP. For validation of gene clusters, we colored the cells in the UMAP according to mean expression of these genes. For each cell, the sum of the TPMs for the genes of interest were extracted and divided by the number of genes to get an average TPM for this set of genes. The mean expressions for all cells were then converted to z-scores for plotting.

For the RNA velocity analysis, velocyto version 0.17 was run on the BAM files provided by Haber et al. The resulting loom files were loaded into Python and analyzed using scanpy version 1.9.1 and scvelo version 0.2.4 (refs. ^61,62^). The cells from the different batches were merged, and the spliced and unspliced layers were pre-processed using scanpy.pp.filter_and_normalize (min_shared_counts = 20 and n_top_genes = 2,000) and scanpy.pp.moments (n_pcs = 30 and n_neighbors = 30). Then, RNA velocity was estimated using scanpy.tl.velocity with the model ‘stochastic’ and plotted on the UMAP computed previously using scanpy.pl.velocity_embedding_stream with smooth = 0.8 and min_mass = 3. Moreover, the pseudotime was estimated using diffusion pseudotime (DPT)^63^. The dataset was normalized and log-transformed, and genes and cells were filtered (min_cells = 10, min_genes = 100). The root of the dataset was set to the stem cell cluster, after which scanpy.tl.dpt was run with default parameters to obtain the pseudotime of each cell, which were plotted on the UMAP.

### Enhancer DMR calling and validation for ESCs

Potential enhancer regions were called by filtering all genome-wide DCM sites using BEDTools version 2.29.2 (ref. ^55^) based on the following filters: (1) not overlapping any known genes from Ensembl version 98, (2) more than 1-kb distance to the closest gene and (3) not overlapping any repeat region from the UCSC RepeatMasker track. For each of the resulting 4.1 million sites, the number of overlapping DCM reads was counted and normalized to TPM using the total number of DCM reads per sample. Differentially methylated sites between the dox-treated and control ESC samples were selected based on a Mann–Whitney test (*P* < 0.05) and fold change ≥4. Enhancer sites were merged into enhancer regions when they were less than 500 bp apart. The genomic regions around these candidate enhancer sites were visualised using deepTools version 3.5.0 (ref. ^64^) by plotting the TPM-normalized tracks. Moreover, overlap with publicly available ChIP-seq datasets from ESCs (ENCSR000CGN, ENCSR000CMW, ENCSR000CGQ, ENCSR000CGO, ENCSR000CFO, ENCSR000CFN, ENCSR000CFZ, ENCSR779CZG, ENCSR000CCD, ENCSR392DGA and ENCSR000CCC from the ENCODE portal) was plotted. The H3K122ac and H3K64ac tracks from Pradeepa et al.^65^ (SRX1560887, SRX1560888, SRX1560889 and SRX1560890) were retrieved from NCBI Sequence Read Archive using sra-tools version 11.0. and reanalyzed. Reads were mapped to mm10 using bowtie2 version 2.4.1, after which the BAM files were normalized using the ‘callpeak’ and ‘bdgcmp’ functions of MACS2 version 2.2.7.1 (ref. ^66^). We visualized the differences in DCM peak height by dividing all differentially methylated sites in three equally sized groups based on the average TPM of the dox-treated samples and plotting overlap with ChIP-seq tracks separately.

The closest gene for each candidate enhancer was selected using BEDTools version 2.29.2 with only the genes that were significantly labelled by dox. Significantly labeled genes were grouped in three equally sized groups based on their fold change. The density of enhancers in the 20-kb region around these three gene groups and the non-significant genes were plotted using deepTools version 3.5.0 and a custom Python script. We plotted the normalized DCM count in the +dox samples for the genes close to enhancer DMRs together with the genes close to H3K27Ac peaks as a control. H3K27Ac peaks were downloaded from the ENCODE portal and processed similarly to the DCM DMRs by removing peaks overlapping gene bodies and <1 kb from genes. *P* values were calculated using a one-sided Wilcoxon rank-sum test. For visualization in the genome browser overviews, we extended the enhancer regions with 250 bp in both directions. Enhancers that overlapped after extension were merged into larger enhancer regions.

### Enhancer DMR calling and validation for intestine

For each of the filtered DCM sites, the number of overlapping DCM reads was counted and normalized to TPM. Differentially methylated sites between all dox-treated and control intestine samples were selected based on (1) Mann–Whitney test (*P* < 0.05), (2) fold change ≥4 and (3) minimal ten overlapping reads. The average TPM-normalized tracks per day were used for visualization. We split the DMRs in seven groups based on the day with the maximum DCM signal. For each peak day group separately, the overlap with several ChIP-seq datasets was examined (SRX3920113, SRX3920114, SRX3920117, SRX3920105, SRX3920106, SRX3920107, SRX3920108, SRX5023289 and SRX5023290 from Chen et al.^26^). These datasets were reanalyzed as described for the ESC ChIP-seq data. Moreover, ChIP-seq datasets for H2AZ (SRX2339011, SRX2339012, SRX2339013, SRX2339022, SRX2339023 and SRX2339024 from Kazakevych et al.^35^), H2AK119ub (SRX856956, SRX856957, SRX856959 and SRX856960 from Chiacchiera et al.^31^), H3K27me3 (SRX2339102, SRX2339103, SRX2339104, SRX2339111, SRX2339112 and SRX2339113 from Kazakevych et al.^35^) and ATOH1 from Lo et al.^67^ (SRX1817263, SRX1817257, SRX1817249, SRX1817250, SRX1817251, SRX1817253, SRX1817254, SRX1817252 and SRX1817255) were also reanalyzed and plotted on the enhancer DMRs and the TSS of significantly labeled genes for each peak day separately.

The closest gene for each candidate enhancer was selected using BEDTools version 2.29.2. For both enhancers and their closest genes, we selected the day with the highest average TPM-normalized DCM count as peak day. The average TPM-normalized DCM counts per day were converted to z-scores for each region separately to visualize their patterns over time in heat maps. The density of enhancers per peak day in the 20-kb region around the genes per peak day was plotted using deepTools version 3.5.0 and a custom Python script. We visualized the correlation between peak day of enhancers and closest genes using the number of enhancers in the 3-kb region around each gene. For each combination of enhancer peak day and gene peak day, the normalized number was plotted.

### Percentage of active enhancers labeled in intestine

To address what percentage of active enhancers was labeled by DCM in intestine, active intergenic enhancers were selected based on H3K27ac peaks. Villi ChIP-seq data from Saxena et al.^23^ were re-analyzed as described. The H3K27ac peaks called by MACS2 were filtered similarly to the DCM sites: (1) not overlapping a gene body and (2) >1 kb from a gene body. The intergenic H3K27ac peaks were plotted in a heat map using deepTools version 3.5.0, showing the overlap with several ChIP-seq datasets and the DCM data from −dox samples and the day 1 and day 2 +dox samples. The peaks were ordered according to the overlapping DCM signal ±1 kb of the peak center and split in four equally sized groups based on this ordering. The correlation at the H3K27ac peaks between the different datasets was examined by counting the number of reads overlapping each H3K27ac peak with ≥3 DCM sites. These read counts were normalized for the peak length or the number of DCM sites for the ChIP-seq and DCM datasets, respectively. For each combination of datasets, the Spearman correlation was calculated using these normalized counts.

Peaks were classified as labeled when at least one significant DCM site was overlapping the peak, whereas non-labeled peaks contained no significant DCM site. A set of random controls was generated by randomly permuting the H3K27ac peaks 100 times separately using BEDTools shuffle (excluding genic regions ±1 kb). The distance to the closest significant DCM site was plotted, and the number of overlapping DCM sites was counted. Based on the distance to the closest significant DCM site compared to the random control, H3K27ac peaks with a significant DCM site <750 bp were selected as active enhancers labeled by DCM.

### Motif analysis

To identify TF binding locations at the intestine enhancer regions, a motif analysis was performed using the R package chromVAR^68^. We counted the number of reads overlapping each enhancer region for each sample separately and provided the total number of DCM reads per sample to normalize for the sequencing depth. The candidate enhancer sites were extended with 250 bp in both directions to obtain regions for motif finding. The package motifmatchr was used to find motifs within these regions based on the motifs retrieved from the JASPAR 2018 database using a *P* value cutoff of 4 × 10^−5^ (ref. ^69^). The motif scores calculated by chromVAR were downloaded and further analyzed in Python. For each motif, the motif scores for all samples were plotted against the z-score of normalized gene DCM counts of the corresponding TF. The Pearson correlation between both scores was calculated, and genes with a correlation >0.3 or <−0.3 were retained for plotting. For motifs that are co-bound by two or more TFs, the gene with the highest correlation with the motif scores was used.

We compared the temporal patterns of the motifs occurring >100 times to the temporal patterns of the genes themselves. Motifs with an early or late maximum temporal signal strength were selected based on the following criteria: (1) the highest signal strength was found early (day 1 or 2) or late (day 6 or 8), respectively, and (2) the second or third highest strength also occurred early or late. The temporal patterns of the early and late motifs and their related genes were plotted separately. Candidate TFs for enterocytes and ISCs were selected as TFs with a maximum DCM gene body accumulation as well as maximum motif proportion at days 1–2 and days 6–8, respectively.

### WGBS

The following experiments were performed at GenomeScan B.V. following SOP176 draft version 8. (1) Concentration was determined using the QuantIT BR Kit. (2) To each normalized sample, 5 µl of 100× diluted Lambda Conversion Control (CC; SeqCap Epi Accessory Kit) was added. (3) The combined DNA + CC was fragmented to ~300 bp. (4) To >235 ng of DNA, 0.75 ng of GS spike in bisulfite conversion oligo (BCO) was added. (5) Library prep was performed with the NEBNext Ultra II DNA Kit and dsDNA adapters from Integrated DNA Technologies. (6) Bisulfite conversion was performed using the EZ-96 DNA Methylation-Lightning MagPrep Kit. (7) The converted libraries were amplified using the KAPA HiFi HotStart Uracil+ ReadyMix 2× using ten PCR cycles (samples 6–8, 12 cycles). (8) Concentration of the samples was determined using the QuantIT HS Kit. Size of the libraries was determined using the FA HS Kit. (9) Before the hybridization, the conversion ratio of the BCO control was determined using ddPCR. (10) Clustering and DNA sequencing using the NovaSeq 6000 was performed according to the manufacturerʼs protocols. A concentration of 1.1 nM of DNA was used. Image analysis, base calling and quality check were performed with the Illumina data analysis pipeline RTA 3.4.4 and Bcl2fastq version 2.20.

The unique molecular identifier (UMI) barcodes from each read were added to the read names using UMI-tools version 1.1.2 extract^70^. Trim Galore version 0.6.7 (wrapper of Cutadapt version 1.18 (ref. ^71^)) was run with default settings for adapter and quality trimming. The reads were bisulfite mapped using bismark version 0.23.1 (ref. ^72^) (–pbat and –bowtie2) and deduplicated using deduplicate_bismark based on the barcode information in the read names (–barcode). Methylation calls for all Cs were obtained using bismark_methylation_extractor with the settings –bedGraph and –CX to also consider cytosines in the non-CpG context. Moreover, coverage2cytosine (withv –CX) was run to obtain genome-wide cytosine methylation reports. To obtain DCM methylation-specific bedGraphs, the bedGraph with all Cs was filtered for Cs overlapping DCM sites using BEDTools intersect. We evaluated the correlation between MeD-seq and WGBS. For all genes with at least ten DCM sites, the average WGBS methylation percentage was plotted against the average number of DCM MeD-seq reads (*n* = 3) normalized for the number of DCM sites. Genes active based on MeD-seq analysis were highlighted, and the Spearman correlation was reported.

To understand the efficiency of DCM labeling better, we analyzed how often DCM sites in reads are co-methylated. We selected reads overlapping genes with at least two DCM sites, both with a methylation percentage above 0.0% in all reads. When reads had more than two DCM sites, we focused only on the first and last DCM site. From these reads, we scored how often both sites were methylated, how often either of one was methylated or how often both were unmethylated. Moreover, two control simulated datasets were added to represent a fully unlinked and a fully linked situation. For the fully unlinked situation, we used the same reads as in the dataset but simulated the methylation status of the sites. For both sites separately, we extracted the average methylation percentage from all reads and generated a random number, which is either above (unmethylated) or below (methylated) this number. For the fully linked situation, we also simulated a dataset based on the reads. From the average methylation percentage for both sites, we extracted the lowest percentage as the percentage in which both sites are methylated and again generated a random number to obtain the simulated methylation status. If the sites were simulated to be unmethylated, the difference in average methylation percentages between both sites was used to decide whether one of both sites was methylated.

### CUT&Tag analysis

ISCs were isolated from LGR5-EGFP transgenic mice (LGR5-EGFP: B6-Lgr5tm1(cre/ERT2)Cle/J). Unless stated otherwise, steps were performed at 4 °C and centrifugation for 5 minutes at 300*g* and 4 °C. The entire small intestine was collected, flushed with cold PBS and opened longitudinally, and villi were removed with a glass slide. The intestine was cut into 5-mm pieces and washed four times in cold PBS. After washing, the pieces were incubated twice in 10 mM EDTA for 15 minutes and 90 minutes at 4 °C. After EDTA incubation, crypts were mechanically separated from stromal tissue in cold PBS with a 10-ml serological pipette. The crypts were collected in the supernatant and centrifuged. The pellet was resuspended in advanced DMEM/F12 (ADF) and incubated with DNase for 10 minutes at room temperature. Next, the crypts were filtered through a 70-µm cell strainer and centrifuged for 5 minutes at 80*g* and 4 °C. Crypts were dissociated to single cells in TrypLE Select Enzyme (1×, Gibco, 12563011) for 3 minutes at 37 °C, and cells were disrupted every 60 seconds with a P1000. TrypLE was diluted with ADF, and cells were washed twice with 5% FCS in HBSS. Cells were incubated with antibodies (TER-119, BD Horizon, 563998; CD31, BD Horizon, 563356; CD45, BD Horizon, 563890; CD24-Apc, BioLegend, 101814; CD117-PE, BioLegend, 105808) for 30 minutes at 4 °C and washed twice in 3.5 ml of 5% FCS in HBSS. Cells were filtered through a 40-µM cell strainer before proceeding to FACS. The FACS-sorted cells were centrifuged for 5 minutes at 200*g* and 4 °C and resuspended in CUT&Tag washing buffer.

To study the genome-wide distribution of H2A.Z and H2A.Zac in the ISCs and enterocytes, a CUT&Tag experiment was performed (Kaya-Okur et al.^73^). Whereas, for the ISCs, the whole cells could be used for CUT&Tag, for enterocytes the nuclei had to be isolated due to crossover in animal of origin between FACS (Glut2) and CUT&Tag (H2A.Z and H2A.Zac) antibodies. After the FACS procedure, enterocytes were centrifuged for 5 minutes at 100*g* and 4 °C, and pellet was resuspended in TST buffer (0.5% Tween, 1% BSA, 10 mM Tris-HCl pH 7.5, 1 mM CaCl_2_, 146 mM NaCl and 41 mM MgCl_2_ in MQ). Subsequently, nuclei were collected by centrifuging for 10 minutes at 100*g* and 4 °C. Nuclei were incubated for 5 minutes on ice in 800 µl of TST buffer and centrifuged for 10 minutes at 100*g* and 4 °C. Nuclei were resuspended in CUT&Tag wash buffer.

CUT&Tag was performed following published protocol with minor adaptions^73^. Per condition, 3.5 × 10^4^ ISCs and 1 × 10^5^ enterocyte nuclei were used as input. The same protocol was followed for both cells and nuclei. Samples were incubated O/N with 1:100 primary antibody (rabbit anti-histone H2A.Z, Abcam, ab4174; rabbit anti-acetyl histone H2A.Z, Merck, ABE1363; or rabbit anti-H3K27me3, Cell Signaling Technology, 9733) and for 1 hour with secondary antibody (rabbit α-mouse antibody, Abcam, ab46540). The pA-Tn5 adaptor complex was incubated for 1 hour at room temperature (pA-Tn5 transposase (loaded)) (Diagenode, C01070001). After DNA extraction, pellet was resuspended in 10 µl of 0.1 mM EDTA by vortex. Sequencing librabries were prepared with the published CUT&Tag amplification method. The libraries were sequenced on an Illumina HiSeq 2500 sequencer, and paired-end clusters were generated of 50 bases in length. The reads were mapped to mm10 using bowtie2 version 2.4.1 (–end-to-end–very-sensitive–no-mixed–no-discordant–phred33 -I 10 -X 700). CPM-normalized bigWig tracks were made using deepTools version 3.5.0 bamCoverage (-bs 1–normalizeUsing CPM) for visualization.

### Reporting summary

Further information on research design is available in the Nature Portfolio Reporting Summary linked to this article.

## Online content

Any methods, additional references, Nature Portfolio reporting summaries, source data, extended data, supplementary information, acknowledgements, peer review information; details of author contributions and competing interests; and statements of data and code availability are available at 10.1038/s41587-023-01683-1.

### Supplementary information

Supplementary Information Supplementary Figs. 1–10, Source Data for Supplementary Fig. 1 and descriptions of Supplementary Tables 1–6.

Reporting Summary

Supplementary Tables 1–6.

## Data Availability

MeD‐seq, RNA-seq, WGBS and CUT&Tag sequencing data are deposited at the National Center for Biotechnology Information with accession number PRJNA615329 (ref. ^74^). In addition, the following datasets were downloaded and used for analysis. From the ENCODE portal (https://www.encodeproject.org), mouse ESC: ENCSR000CCC, ENCSR000CMW, ENCSR000CFO, ENCSR000CCD, ENCSR000CGN, ENCSR000CGO, ENCSR000CFZ, ENCSR779CZG, ENCSR392DGA, ENCSR000CGQ, ENCSR000CFN and ENCSR000CGR; mouse intestine: ENCSR159RVN, ENCSR198ACZ, ENCSR311VKI, ENCSR642VYW, ENCSR389EYR, ENCSR483KOD, ENCSR000CEE and ENCSR079GOY. From the Gene Expression Omnibus, GSE83394, and from the Sequence Read Archive using sra-tools version 2.11.0: SRX1560887, SRX1560888, SRX1560889, SRX1560890, SRX3920113, SRX3920114, SRX3920117, SRX3920105, SRX3920106, SRX3920107, SRX3920108, SRX5023289, SRX5023290, SRX2339011, SRX2339012,SRX2339013, SRX2339022, SRX2339023, SRX2339024, SRX856956, SRX856957, SRX856959, SRX856960, SRX2339102, SRX2339103, SRX2339104, SRX2339111, SRX2339112, SRX2339113, SRX1817263, SRX1817257, SRX1817249, SRX1817250, SRX1817251, SRX1817253, SRX1817254, SRX1817252 and SRX1817255. scRNA-seq data: GSE92332 and GSE46980.

## Code availability

In general, custom Python scripts are available upon reasonable request with exceptions for scripts concerning processing of raw MeD-seq data, which are limited under licensing agreements between Erasmus Medical Center and commercial partners. However, scripts regarding downstream analysis of MeD-seq data are available upon reasonable request.
