# Supplementary material for: Retrospective analysis of enhancer activity and transcriptome history
Source: Nat Biotechnol. 2023 Feb 23;41(11):1582–92. doi: 10.1038/s41587-023-01683-1 (PMC10635829; doi:10.1038/s41587-023-01683-1)
Supplement: Supplementary file 1 — Supplementary Figs. 1–10, Source Data for Supplementary Fig. 1 and descriptions of Supplementary Tables 1–6. [file 41587_2023_1683_MOESM1_ESM.pdf]

nature biotechnology

Article https://doi.org/10.1038/s41587-023-01683-1

Retrospective analysis of enhancer activity and transcriptome history

In the format provided by the authors and unedited

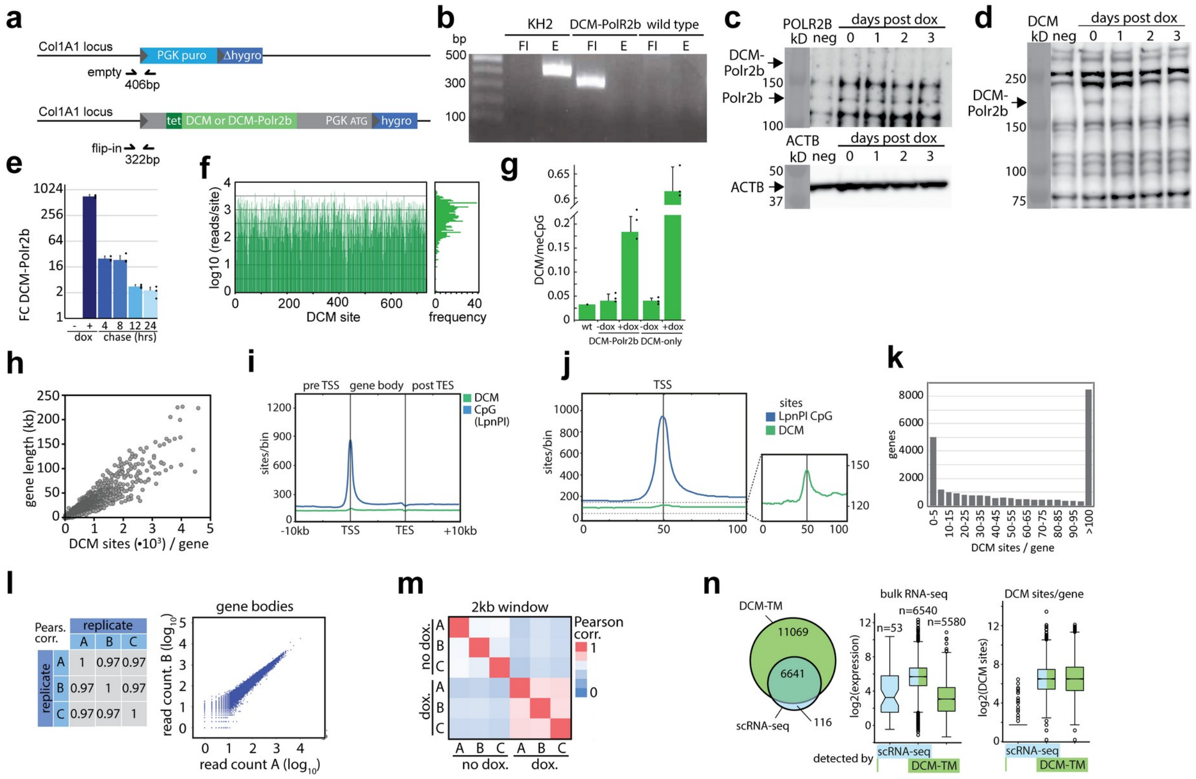

Supplementary Figure 1

(a) The DCM-Polr2b fusion gene was introduced in the Col1A1 locus by Flipase mediated insertion. (b) PCR with primers for flip-in (FI) and empty (E) indicated in (a) verifying proper integration of the transgene (representative image shown from n=2 replicates). (c,d) Western blotting analysis detecting POLR2B (c, top panel), ACTB (c, bottom panel) and DCM (d) on DCM-Polr2b ES cells 5 days after induction with dox and 1,2 and 3 days after removal of dox (representative image shown (c and d) from n=3 replicates). (e) qRT-PCR analysis detecting DCM-POLR2B transcript levels in ES cells at different timepoints (hours) after removal of dox (average with SEM plotted, n=3 per condition). (f) Read coverage per DCM site from mouse BAC, frequency of coverage plotted on the right. (g) Ratio of DCM/CpG methylation in wild type (WT), DCM-Polr2b and DCM-only ESCs -dox and +dox (average with SEM plotted, n=3 per condition). (h) Scatter plot displaying gene length and number of DCM sites per gene. (i) Gene meta-analysis showing binned distribution of DCM and CpG sites. (j) Zoom of the TSS region shown in (i). (k) Histogram showing distribution of DCM sites per gene. (l) Pearson correlation analysis comparing gene body DCM methylation in three replicate experiments with 5 days dox induced DCM-Polr2b ES cells. Right panel displays one example of a per gene body comparison. (m) Pearson correlation analysis comparing DCM methylation in 2kb genome wide windows between three replicate experiments with and without a 5 day dox induction of DCM-Polr2b ES cells. (n) Overlap of genes that are labelled by DCM methylation (green) and

detected by scRNA-seq (more than 5 reads, blue), gene counts in Venn diagram. The boxplots show log2 expression of bulk RNA-seq and log2 DCM sites for scRNA-seq specific genes, DCM-TM specific genes and genes detected by both DCM-TM and scRNA-seq (boxplots show 25th percentile, median and 75th percentile, with the whiskers spanning 97% of the data).

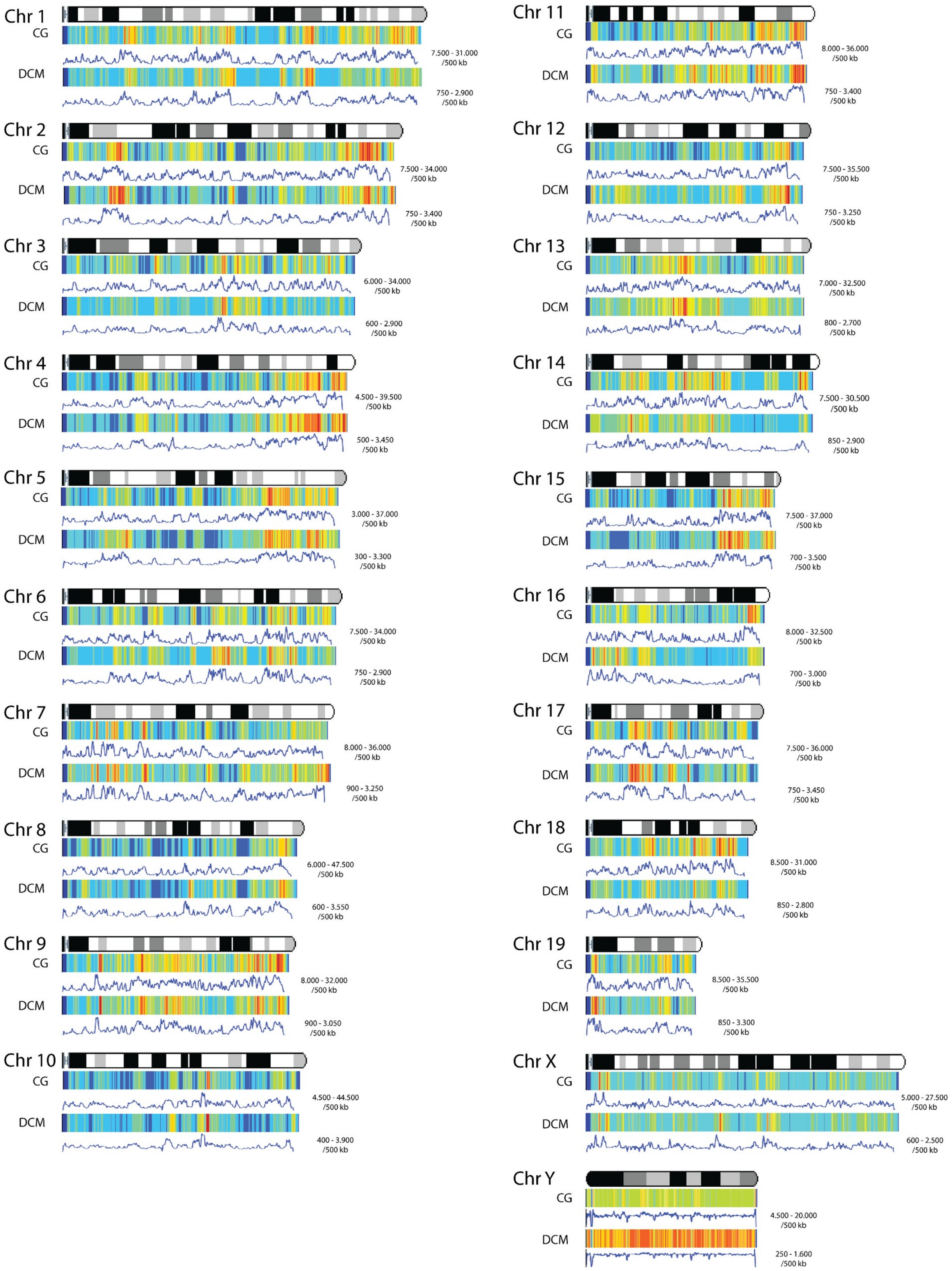

Supplementary Figure 2

Genome wide overview of DCM and CpG density.

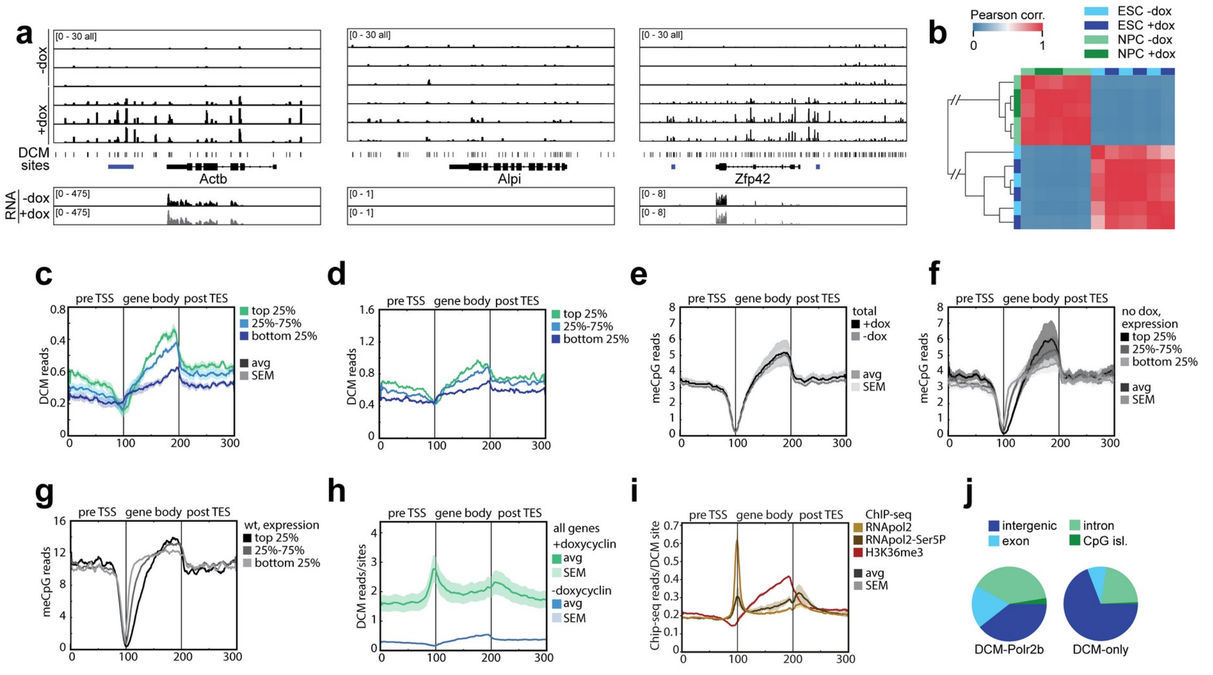

Supplementary Figure 3

(a) Genome browser view of DCM specific MeD-seq reads (n=3) and RNA-seq reads (average of n=3) in the *Actb*, *Alpi* and *Zfp42* loci (intergenic DMRs are indicated in blue). (b) Pearson correlation between replicates of ESCs with (dark blue, n=3) and without dox (light blue, n=3) and NPCs with (dark green, n=2) and without dox (light green, n=3). Part of the dendrogram has been truncated for visualization. (c) Gene meta-analysis showing binned distribution of DCM reads in the top 25%, 25%-75% and bottom 25% expressed genes in DCM- Polr2b ESCs in the absence of dox. The average (avg) and standard error of the mean (SEM) are depicted with a darker line and lighter region, respectively. (d) As in (c) for wild type ESCs (n=1). (e) Gene meta-analysis showing binned distribution of all CpG reads before and after dox treatment (5 days). (f) Gene meta-analysis showing binned distribution of CpG reads in the top 25%, 25%-75% and bottom 25% expressed genes in DCM- Polr2b ESCs in the absence of dox. (g) As in (f) for wild type ESCs (n=1). (h) Gene meta-analysis showing binned distribution of DCM reads overlapping the gene body before (blue) and after (green) 5 days of dox treatment in DCM- Polr2b ESCs. (i) Gene meta-analysis showing binned distribution of RNApol2, RNApol2-Ser5P, and H3K36me3 ChIP-seq reads. (j) Genome wide distribution split by genomic feature of DCM reads in DCM-Polr2b and DCM-only ES cells after a 5 day dox induction.

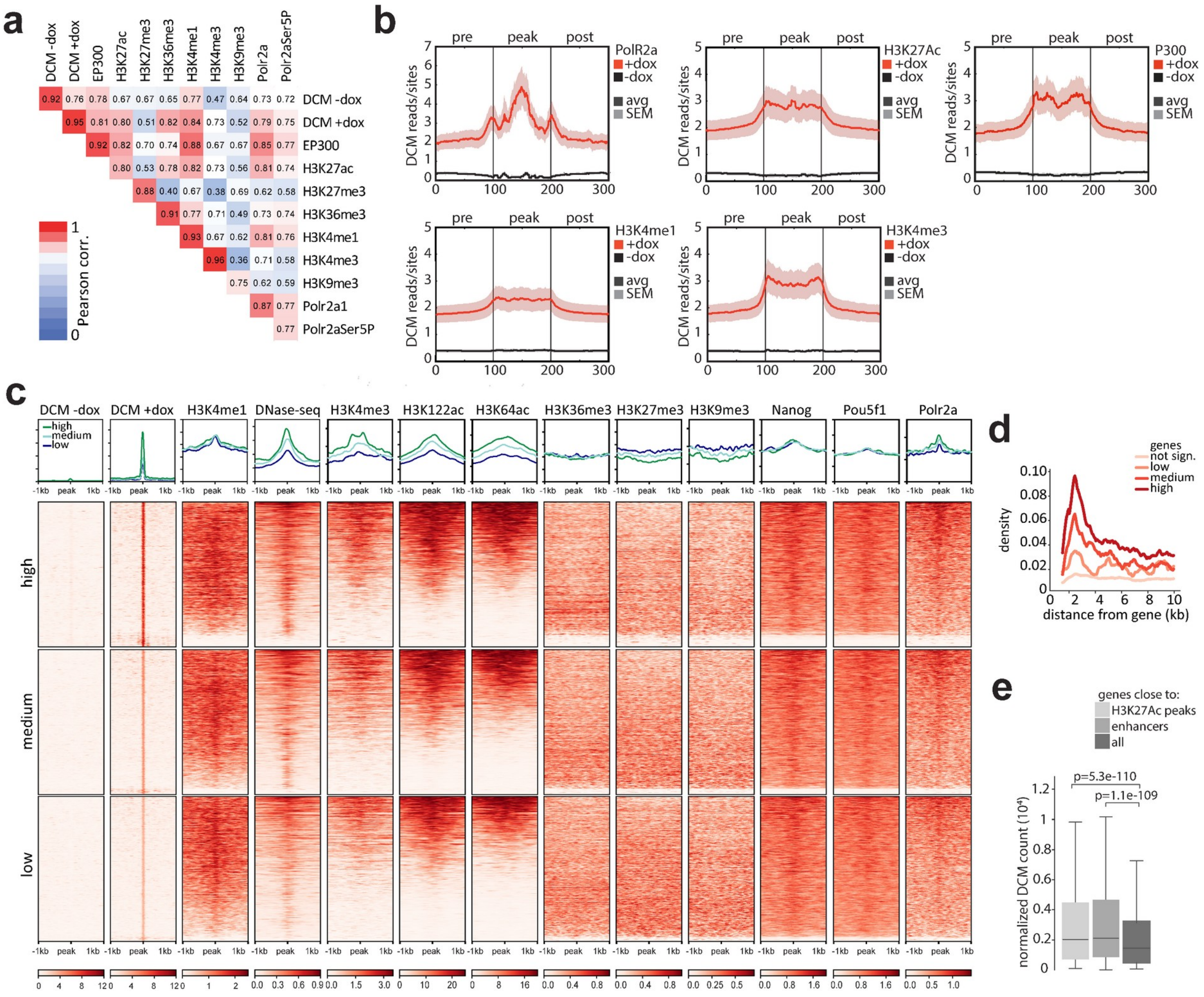

Supplementary Figure 4

a) Pearson correlation analysis comparing DCM and ChIP-seq read count distribution. (b) Enhancer meta-analysis showing binned distribution of DCM read counts over Polr2a, H3K27Ac, P300, H3K4me1 and H3K4me3 positive genomic regions and 1kb proximal and distal flanking regions. (c) Heatmap showing ChIP-seq overlap with the regions around enhancer DMRs. DMRs are split in three clusters based on the normalized number of reads for +dox. Each profile plot has the same y-axis range as its corresponding heatmap. (d) Density plot showing the number of enhancer DMRs in the 10kb region around genes that were either not significantly labelled by DCM or genes split in three clusters based on fold change between +dox and –dox (e) Normalized DCM count of +dox samples for genes close to the enhancer DMRs, close to H3K27ac peaks and all genes. P-values were calculated using a one-sided Wilcoxon rank-sum test ($n_{H3K27ac}$ = 6441, $n_{enhancers}$ = 3107, n_{all} = 11372, boxplots show 25th percentile, median and 75th percentile, with the whiskers spanning 97% of the data).

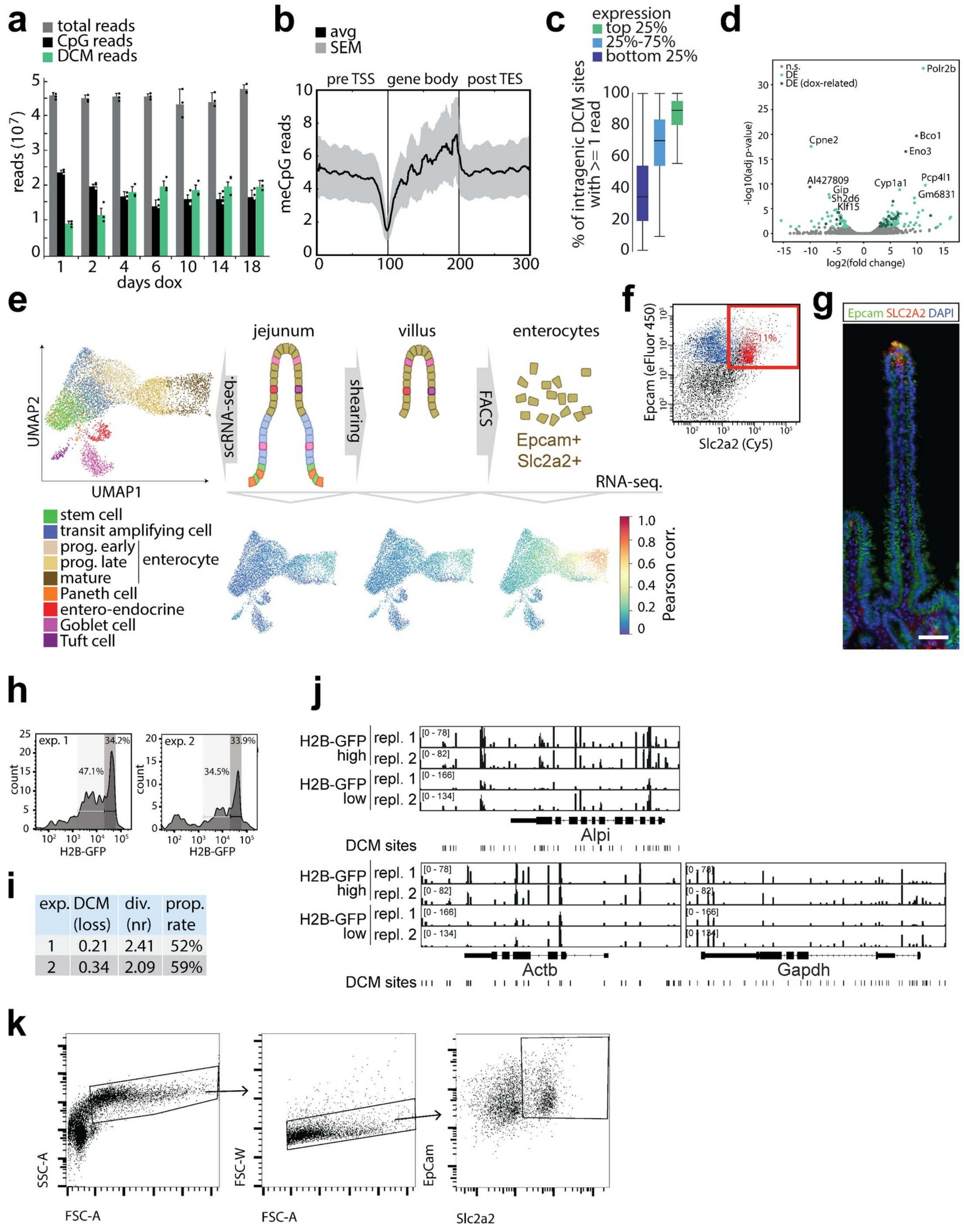

Supplementary Figure 5

(a) Genome wide DCM and CpG methylation at different time points after start of dox induction (average with SEM plotted, n=3 per condition). (b) Gene meta-analysis showing binned distribution of all CpG reads in the absence of dox. (c) Boxplot showing the percentage of intragenic DCM sites that contain DCM methylation data measured by MeD-seq, separated in three groups of genes based on RNA-seq expression levels (n=3333 top 25%, n=6220 25-75%, n=2770 bottom 25%,

boxplots show 25th percentile, median and 75th percentile, with the whiskers spanning 97% of the data). (d) Differential gene expression analysis of jejunum epithelium comparing DCM-Polr2b:m2rtTA and m2rtTA-only mice with and without 5 days doxycycline (P-values calculated using the DESeq2 Wald test). Differentially expressed genes between dox treated epithelium of DCM-Polr2b:m2rtTA and m2rtTA-only are highlighted in dark green (dox-related DE). (e) Overview of the experimental procedure to isolate SLC2A2 expressing enterocytes. Villi were isolated from intestinal epithelium of jejunum followed by FACS isolation of EpCAM/SLC2A2 positive cells. Left panel shows UMAP of scRNA-seq data colored according to annotation as specific cell types, bottom panels show Pearson correlation analysis of bulk RNA-seq data of total epithelial, villi and EpCAM/SLC2A2 positive fractions with scRNA-seq data. (f) FACS analysis of intestinal epithelial cells, EpCAM/SLC2A2 positive cells are highlighted in red. (g) Immuno-cytochemistry detecting EpCAM (FITC) and SLC2A2 (Texas red, DNA in DAPI, representative image shown from $n \geq 5$ replicates, scale bar: 50μm). (h) FACS analysis showing histograms of H2B-GFP expression in EpCAM/SLC2A2 positive enterocytes (two independent experiments). (i) Quantification of H2B-GFP and DCM loss over a 3-day chase period and calculated propagation rate. (j) Genome browser view of DCM specific MeD-seq reads in *Alpi, Actb*, *Alpi* and *Gapdh* loci in two replicates of H2B-GFP high and H2B-GFP low sorted cell populations. (k) FACS sorting gating strategy to isolate EpCAM/SLC2A2 positive cells.

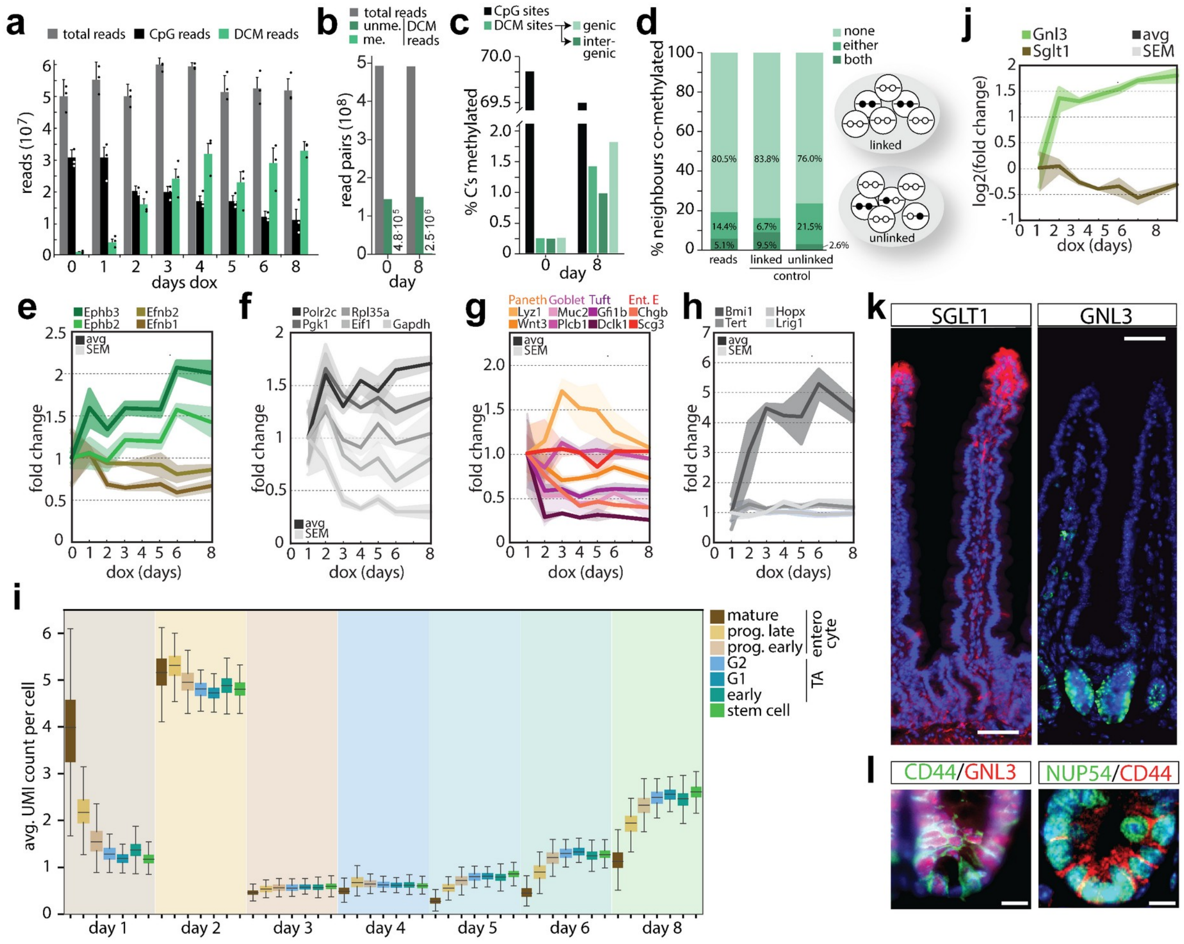

Supplementary Figure 6

(a) Genome wide DCM and CpG methylation levels at different time points after start of dox induction (average with SEM plotted, n=3 per condition). (b) Number of WGBS read pairs sequenced in total, pairs containing unmethylated DCM sites only and pairs containing at least one methylated DCM site (bars with low numbers are annotated, n=1). (c) Percentage of C's methylated in WGBS data from untreated and 8 day dox treated enterocytes (reported separately for C's overlapping CpG, DCM, intergenic DCM and genic DCM sites). (d) Percentage of two neighbouring DCM sites in reads overlapping active genes that are both unmethylated, either methylated or both methylated. Two control simulated datasets are added showing expected percentages for fully linked and fully unlinked situations (schematic examples of both shown in right panel), representing whether DCM labelling is fully random or neighbouring sites have a higher chance of both being methylated, respectively. (e) DCM labelling (relative to total and normalized to T=1d) of *Ephb2, Ephb3, Efnb1*, and *Efnb2* genes, (f) of the ubiquitously expressed *Polr2c, Pgk1, Rpl35a, Eif1* and *Gapdh* genes, (g) of Paneth (*Lyz1*, *Wnt3*), Goblet (*Muc2*, *Plcb1*), Tuft (*Gfi1b*, *Dclk1*) and entero-endocrine (*Chgb*, *Scg3*) markers, (h) and of +4-cell marker genes *Bmi1*, *Tert*, *Hopx* and *Lrig1* at different time points after

start of dox treatment. (i) Normalized UMI count distribution per cell type of genes with an indicated DCM labelling peak time point, showing a shift from enterocyte to ISC specific gene expression and overall increased expression of genes at day 2 (n=822 mature, n=404 prog. late, n=829 prog. early, n=410 G2, n=408 G1, n=665 early, n=1267 stem cell, boxplots show 25th percentile, median and 75th percentile, with the whiskers spanning 97% of the data). (j-k) DCM labelling (j) and validation by immuno-cytochemistry (k) of SGLT1 and GNL3 expression (FITC, DNA is DAPI stained, representative image shown from n=3 (SGLT1) or n≥5 (GNL3) replicates, scale bar: 50μm). (l) Immunocytochemistry detecting GNL3 and NUP54 in combination with CD44 (DNA is DAPI, representative image shown from n≥5 replicates, scale bar: 16μm).

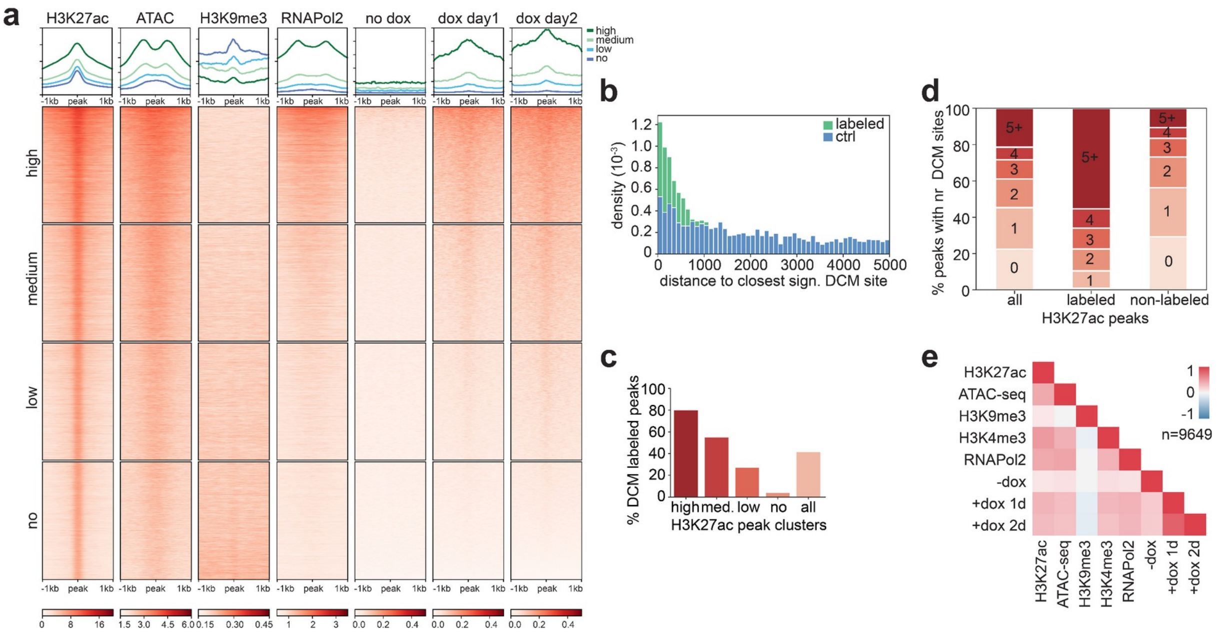

Supplementary Figure 7

(a) Heatmap showing the overlap of villi H3K27ac peaks with several villi ChIP-seq and DCM datasets. All ChIP-seq datasets were generated from villi samples [Saxena et al. 2017] and the DCM data from -dox samples and the day 1 and day 2 +dox samples are shown. H3K27ac peaks are ordered according to overlapping DCM signal ±1kb of the peak center and split in four equally sized clusters based on this ordering (labelled as no, low, medium or high DCM-TM signal). Each profile plot has the same y-axis range as its corresponding heatmap. (b) Histogram with the distance to the closest significant DCM site for both the DCM labeled H3K27ac peaks (*i.e.* peaks with ≥ 1 significant DCM site) and random controls based on 100 sets of reshuffled H3K27ac peaks. Density for each 100 bp bin up to 5kb is shown. (c) Percentage of H3K27ac peaks that are labelled by DCM (*i.e.* peaks with ≥ 1 significant DCM site <750 bp from peak). The H3K27ac peaks are split in four clusters based on H3K27ac intensity and related to all peaks. (d) Barplot showing the number of DCM sites overlapping each peak. The percentages with each number of sites are shown for all peaks, the labeled peaks (*i.e.* peaks with ≥ 1 significant DCM site) and non-labeled peaks (*i.e.* peaks without significant DCM site). (e) Correlation heatmap showing the spearman correlation at the H3K27ac peaks between the different ChIP-seq and DCM datasets shown in (a). The number of reads overlapping each H3K27ac peak with ≥ 3 DCM sites were normalized for the peak length or the number of DCM sites for the ChIP-seq and DCM datasets, respectively.

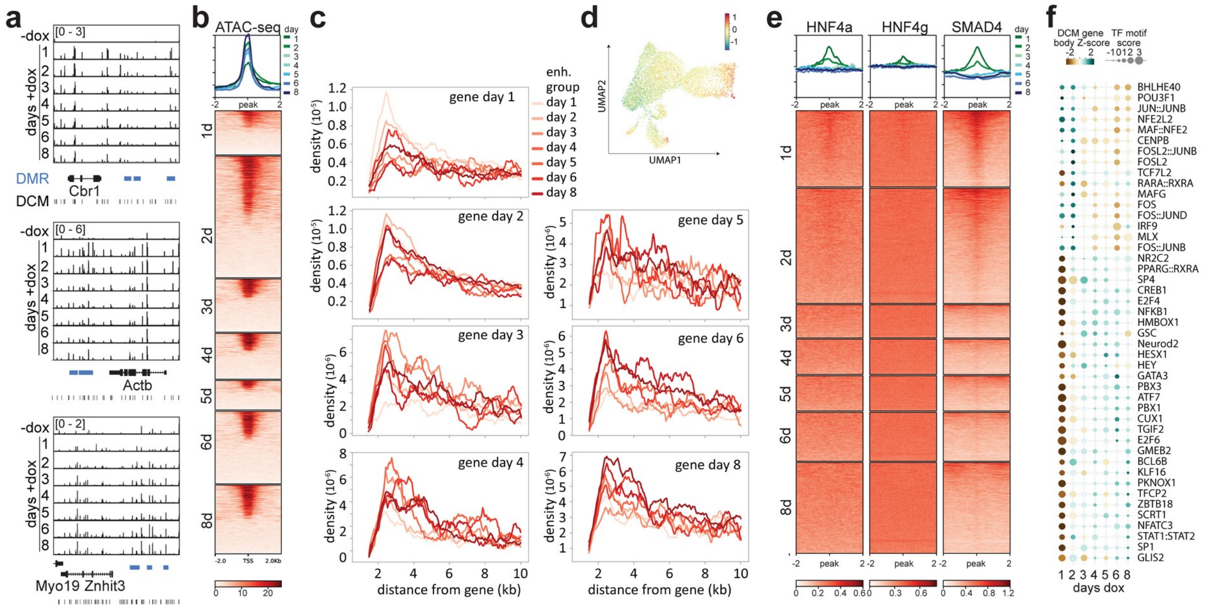

Supplementary Figure 8

(a) Genome browser view showing DCM labelling of enterocyte specific (*Cbr1*), ubiquitous (*Actb*) and ISC specific (*Znhit3*) genes with enhancers (in blue) nearby showing similar behaviour in time (average of n=3). (b) Heatmap showing ATAC-seq overlap with the regions around TSS of genes peaking at different days of dox induction. (c) Density plot showing the number of enhancer DMRs per peak day in the 10kb region around genes split in clusters based on peak timing of gene body DCM labelling. (d) Differential enrichment of genes peaking on day 2 split by having relatively more enhancers peaking on day 2 or day 8 nearby; difference in gene expression plot on UMAP showing higher expression of genes linked to day 2 enhancers in enterocytes. (e) Heatmap showing ChIP-seq overlap for HNFa, HNFg and SMAD4 with regions around enhancer DMRs. Enhancers were split in clusters based on the maximum day of DCM accumulation. (f) Combined analysis of motif enrichment and DCM gene body labelling dynamics of TFs displaying a negative correlation in time.

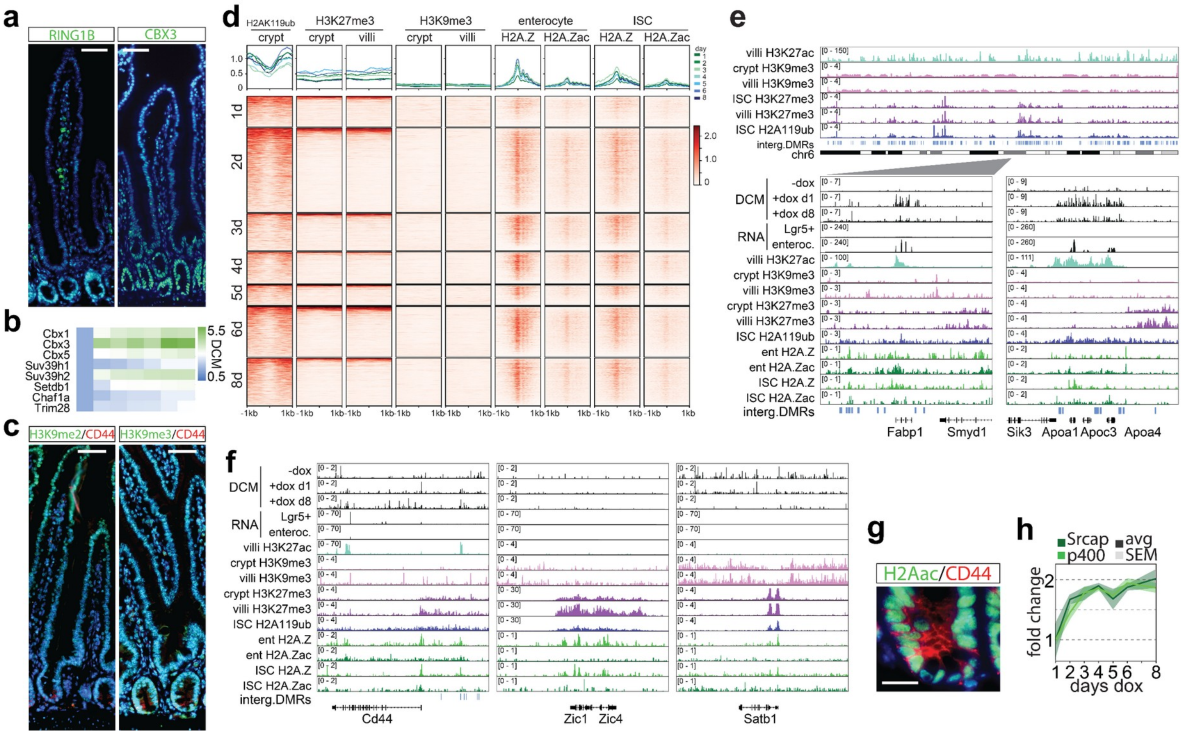

Supplementary Figure 9

(a) Immunocytochemistry detecting RING1B and CBX3 (FITC) (DNA=DAPI, representative image shown from n≥5 replicates, scale bar: 50μm). (b) Temporal behaviour of DCM methylation (normalized to t=1 day) of genes encoding proteins involved in establishment and maintenance of constitutive heterochromatin. (c) Immunocytochemistry detecting H3K9me2 and H3K9me3 (FITC) in combination with CD44 (Texas Red, representative image shown from n≥5 replicates, scale bar: 50μm). (d) Heatmap showing ChIP-seq overlap in enterocytes, ISC, crypt and villi for H2A119ub, H3K27me3, H3K9me3, H2A.Z and H2A.Zac with regions around the TSS. Genes were split in clusters based on the maximum day of DCM accumulation and ordered according to H3K27ac and ATAC-seq enrichment. (e) Genome browser view of DCM specific MeD-seq reads (average of n=3), RNA-seq (+/- dox, average of n=3) and ChIP-seq and Cut&Run tracks in chromosome 6 and *Fabp1* and *Apoa* loci. (f) As (e) for *Cd44*, *Satb1* and *Zic1* loci. (g) Combined detection of H2Aac (FITC) and CD44 (Texas Red) in the intestinal crypt (DNA=DAPI, representative image shown from n≥5 replicates, scale bar: 16μm). (h) DCM labelling (fold change in DCM reads relative to total and normalized to T=1d) of *Srcap* and *P400*.

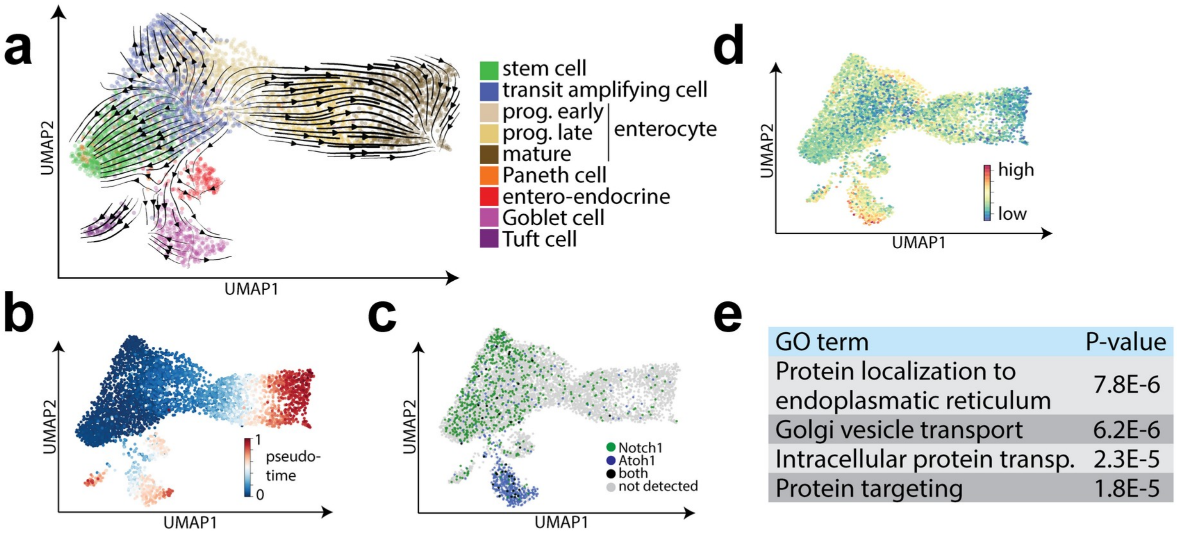

GO term	P-value
Protein localization to endoplasmatic reticulum	7.8E-6
Golgi vesicle transport	6.2E-6
Intracellular protein transp.	2.3E-5
Protein targeting	1.8E-5

Supplementary Figure 10

(a) RNA velocity analysis showing predicted cell fate transitions on the UMAP shown in Fig 3g. (b) Diffusion pseudotime predicted by DPT showing the pseudo-temporal ordering of cells. (c) UMAP displaying cells in which only Notch1 or Atoh1 was detected and cells where both genes or no gene was detected (plotted in UMAP shown in Fig 3g). (d-e) Genes peaking at days 3-5 and significantly higher expression in scRNA-seq for goblet, tuft, entero-endocrine or Paneth cells compared to ISC, early progenitor, late progenitor and mature enterocytes. For this list of genes, the enrichment in the scRNA-seq data is plotted in the UMAP (d) and the enriched GO terms are shown (p-values calculated using a Fisher's Exact test) (e).

Supplementary Table 1

Sequencing statistics.

Supplementary Table 2

Overview of gene body DCM counts in uninduced and induced ES cells and enterocytes.

Supplementary Table 3

Overview of intergenic differentially methylated DCM sites in uninduced and induced ES cells and enterocytes.

Supplementary Table 4

Motif analysis on intergenic DMRs at different timepoints of dox induction in enterocytes.

Supplementary Table 5

GO analysis on top 200 normalized fold enriched GO terms for each set of genes peaking at day 1 and day 8.

Supplementary Table 6

KEGG pathway analysis on differential DCM labelling of genes upon dox induction.

Uncropped Supplemental Figure 1b

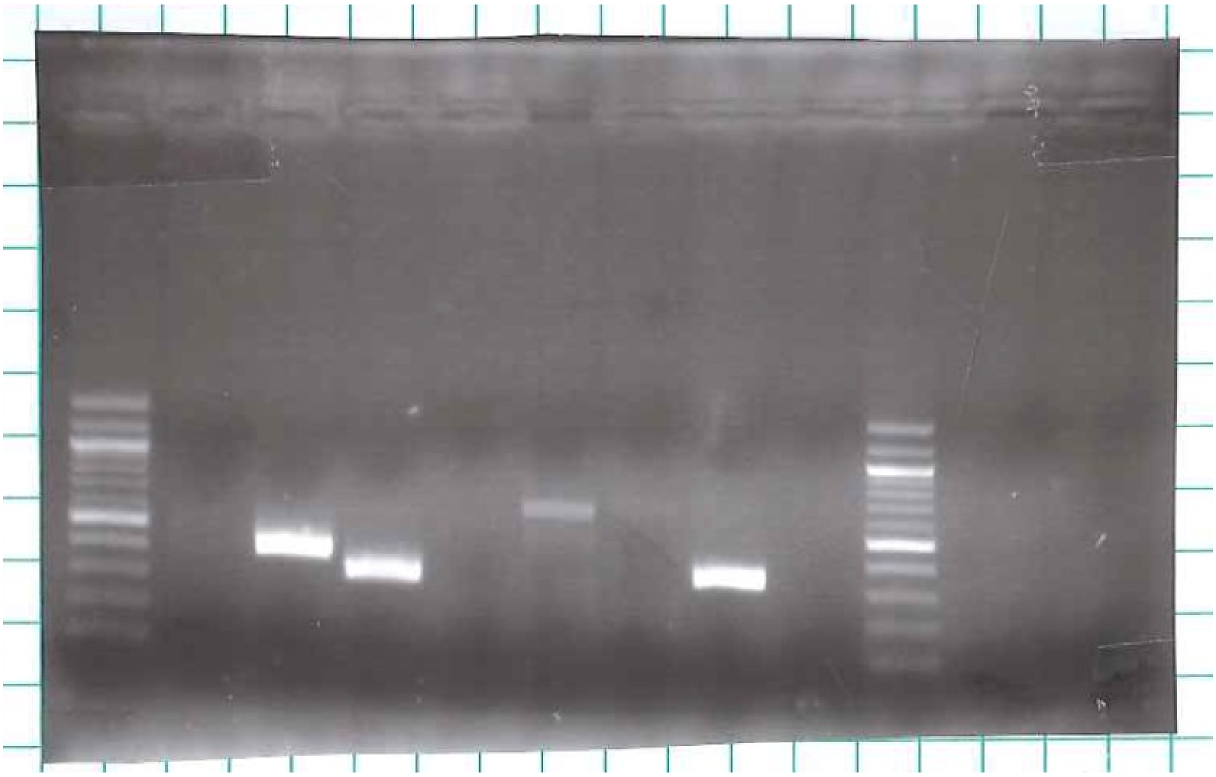

Uncropped Supplemental Figure 1c

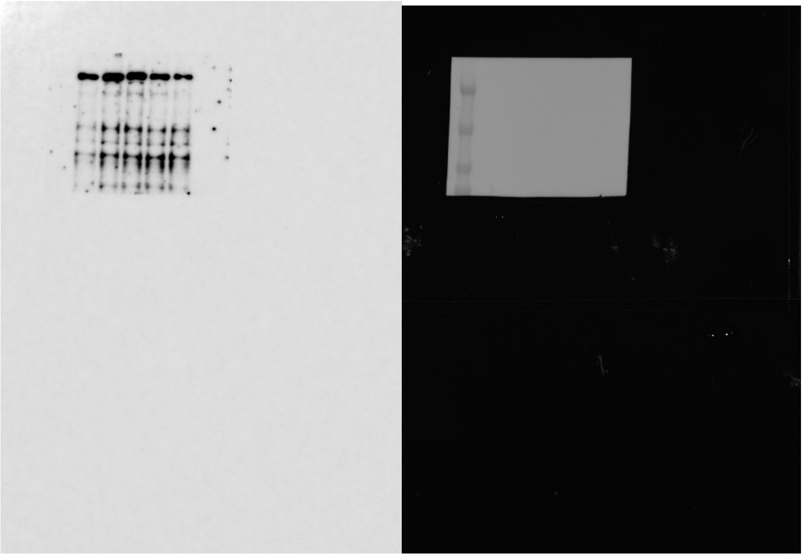

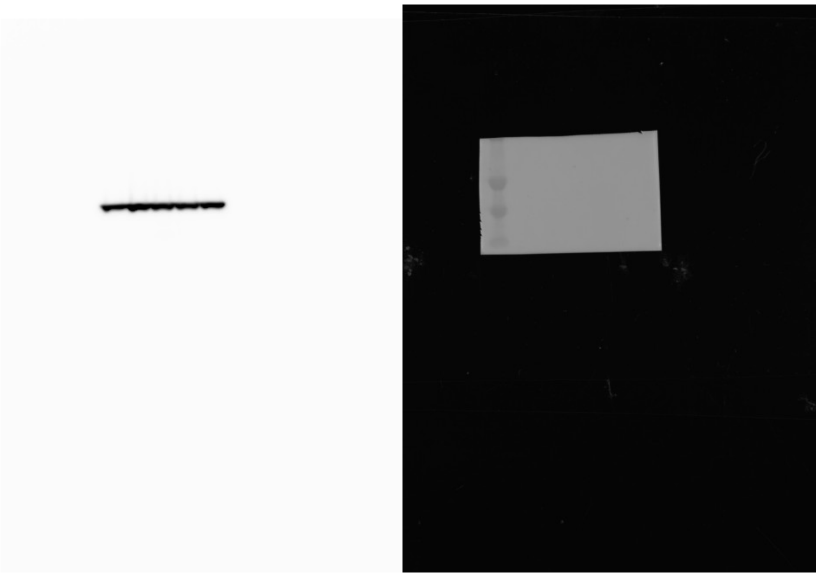

Uncropped Supplemental Figure 1d

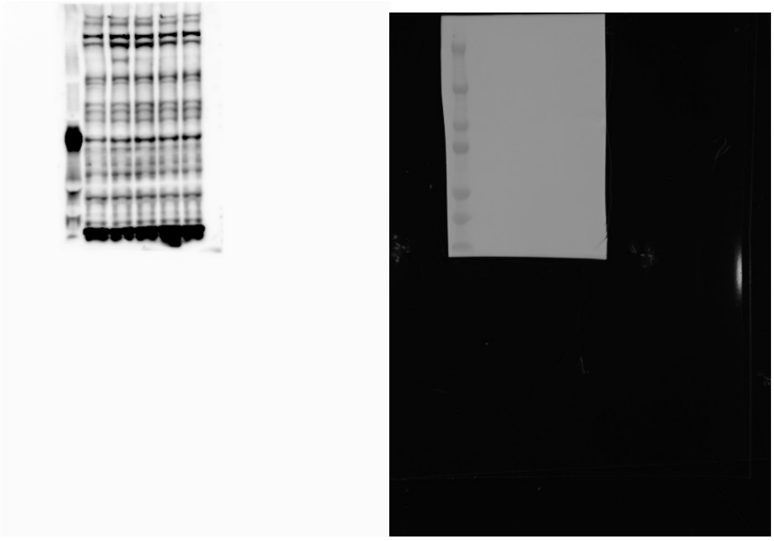

References

1 Bradner, J. E., Hnisz, D. & Young, R. A. Transcriptional Addiction in Cancer. *Cell* **168**, 629-643, doi:10.1016/j.cell.2016.12.013 (2017).

2 Lee, T. I. & Young, R. A. Transcriptional regulation and its misregulation in disease. *Cell* **152**, 1237-1251, doi:10.1016/j.cell.2013.02.014 (2013).

3 Stadhouders, R., Filion, G. J. & Graf, T. Transcription factors and 3D genome conformation in cell-fate decisions. *Nature* **569**, 345-354, doi:10.1038/s41586-019-1182-7 (2019).

4 Alemany, A., Florescu, M., Baron, C. S., Peterson-Maduro, J. & van Oudenaarden, A. Whole-organism clone tracing using single-cell sequencing. *Nature* **556**, 108-112, doi:10.1038/nature25969 (2018).

5 Bowling, S. *et al.* An Engineered CRISPR-Cas9 Mouse Line for Simultaneous Readout of Lineage Histories and Gene Expression Profiles in Single Cells. *Cell* **181**, 1693-1694, doi:10.1016/j.cell.2020.06.018 (2020).

6 Herman, J. S., Sagar & Grun, D. FateID infers cell fate bias in multipotent progenitors from single-cell RNA-seq data. *Nat Methods* **15**, 379-386, doi:10.1038/nmeth.4662 (2018).

7 Schiebinger, G. *et al.* Optimal-Transport Analysis of Single-Cell Gene Expression Identifies Developmental Trajectories in Reprogramming. *Cell* **176**, 1517, doi:10.1016/j.cell.2019.02.026 (2019).

8 Beumer, J. & Clevers, H. Cell fate specification and differentiation in the adult mammalian intestine. *Nat Rev Mol Cell Biol*, doi:10.1038/s41580-020-0278-0 (2020).

9 Barker, N. *et al.* Identification of stem cells in small intestine and colon by marker gene Lgr5. *Nature* **449**, 1003-1007, doi:10.1038/nature06196 (2007).

10 Tetteh, P. W. *et al.* Replacement of Lost Lgr5-Positive Stem Cells through Plasticity of Their Enterocyte-Lineage Daughters. *Cell Stem Cell* **18**, 203-213, doi:10.1016/j.stem.2016.01.001 (2016).

11 Yan, K. S. *et al.* Intestinal Enteroendocrine Lineage Cells Possess Homeostatic and Injury-Inducible Stem Cell Activity. *Cell Stem Cell* **21**, 78-90 e76, doi:10.1016/j.stem.2017.06.014 (2017).

12 Yu, S. *et al.* Paneth Cell Multipotency Induced by Notch Activation following Injury. *Cell Stem Cell* **23**, 46-59 e45, doi:10.1016/j.stem.2018.05.002 (2018).

13 Baron, C. S. & van Oudenaarden, A. Unravelling cellular relationships during development and regeneration using genetic lineage tracing. *Nat Rev Mol Cell Biol* **20**, 753-765, doi:10.1038/s41580-019-0186-3 (2019).

14 Clark, S. J., Harrison, J. & Frommer, M. CpNpG methylation in mammalian cells. *Nat Genet* **10**, 20-27, doi:10.1038/ng0595-20 (1995).

15 Beard, C., Hochedlinger, K., Plath, K., Wutz, A. & Jaenisch, R. Efficient method to generate single-copy transgenic mice by site-specific integration in embryonic stem cells. *Genesis* **44**, 23-28, doi:10.1002/gene.20180 (2006).

16 Boers, R. *et al.* Genome-wide DNA methylation profiling using the methylation-dependent restriction enzyme LpnPI. *Genome Res* **28**, 88-99, doi:10.1101/gr.222885.117 (2018).

17 Islam, S. *et al.* Quantitative single-cell RNA-seq with unique molecular identifiers. *Nat Methods* **11**, 163-166, doi:10.1038/nmeth.2772 (2014).

18 Arand, J. *et al.* In vivo control of CpG and non-CpG DNA methylation by DNA methyltransferases. *PLoS Genet* **8**, e1002750, doi:10.1371/journal.pgen.1002750 (2012).

19 van Steensel, B. & Henikoff, S. Identification of in vivo DNA targets of chromatin proteins using tethered dam methyltransferase. *Nat Biotechnol* **18**, 424-428, doi:10.1038/74487 (2000).

20 Kim, T. K. *et al.* Widespread transcription at neuronal activity-regulated enhancers. *Nature* **465**, 182-187, doi:10.1038/nature09033 (2010).

21 Haber, A. L. *et al.* A single-cell survey of the small intestinal epithelium. *Nature* **551**, 333-339, doi:10.1038/nature24489 (2017).

22 Lopez-Arribillaga, E. *et al.* Bmi1 regulates murine intestinal stem cell proliferation and self-renewal downstream of Notch. *Development* **142**, 41-50, doi:10.1242/dev.107714 (2015).

23 Saxena, M. *et al.* Transcription factor-dependent 'anti-repressive' mammalian enhancers exclude H3K27me3 from extended genomic domains. *Genes Dev* **31**, 2391-2404, doi:10.1101/gad.308536.117 (2017).

24 Calo, E. & Wysocka, J. Modification of enhancer chromatin: what, how, and why? *Mol Cell* **49**, 825-837, doi:10.1016/j.molcel.2013.01.038 (2013).

25 Kaaij, L. T. *et al.* DNA methylation dynamics during intestinal stem cell differentiation reveals enhancers driving gene expression in the villus. *Genome Biol* **14**, R50, doi:10.1186/gb-2013-14-5-r50 (2013).

26 Chen, L. *et al.* A reinforcing HNF4-SMAD4 feed-forward module stabilizes enterocyte identity. *Nat Genet* **51**, 777-785, doi:10.1038/s41588-019-0384-0 (2019).

27 Ng, A. Y. *et al.* Inactivation of the transcription factor Elf3 in mice results in dysmorphogenesis and altered differentiation of intestinal epithelium. *Gastroenterology* **122**, 1455-1466, doi:10.1053/gast.2002.32990 (2002).

28 Ito, N., Kii, I., Shimizu, N., Tanaka, H. & Takeda, S. Direct reprogramming of fibroblasts into skeletal muscle progenitor cells by transcription factors enriched in undifferentiated subpopulation of satellite cells. *Sci Rep* **7**, 8097, doi:10.1038/s41598-017-08232-2 (2017).

29 Lee, B. K. *et al.* Tgif1 Counterbalances the Activity of Core Pluripotency Factors in Mouse Embryonic Stem Cells. *Cell Rep* **13**, 52-60, doi:10.1016/j.celrep.2015.08.067 (2015).

30 Liu, Y. *et al.* The Transcription Factor ATF7 Controls Adipocyte Differentiation and Thermogenic Gene Programming. *iScience* **13**, 98-112, doi:10.1016/j.isci.2019.02.013 (2019).

31 Chiacchiera, F. *et al.* Polycomb Complex PRC1 Preserves Intestinal Stem Cell Identity by Sustaining Wnt/beta-Catenin Transcriptional Activity. *Cell Stem Cell* **18**, 91-103, doi:10.1016/j.stem.2015.09.019 (2016).

32 Blackledge, N. P. *et al.* Variant PRC1 complex-dependent H2A ubiquitylation drives PRC2 recruitment and polycomb domain formation. *Cell* **157**, 1445-1459, doi:10.1016/j.cell.2014.05.004 (2014).

33 Ferrari, K. J. *et al.* Intestinal differentiation involves cleavage of histone H3 N-terminal tails by multiple proteases. *Nucleic Acids Res* **49**, 791-804, doi:10.1093/nar/gkaa1228 (2021).

34 Giaimo, B. D., Ferrante, F., Herchenrother, A., Hake, S. B. & Borggrefe, T. The histone variant H2A.Z in gene regulation. *Epigenetics Chromatin* **12**, 37, doi:10.1186/s13072-019-0274-9 (2019).

35 Kazakevych, J., Sayols, S., Messner, B., Krienke, C. & Soshnikova, N. Dynamic changes in chromatin states during specification and differentiation of adult intestinal stem cells. *Nucleic Acids Res* **45**, 5770-5784, doi:10.1093/nar/gkx167 (2017).

36 Greenberg, R. S., Long, H. K., Swigut, T. & Wysocka, J. Single Amino Acid Change Underlies Distinct Roles of H2A.Z Subtypes in Human Syndrome. *Cell* **178**, 1421-1436 e1424, doi:10.1016/j.cell.2019.08.002 (2019).

37 La Manno, G. *et al.* RNA velocity of single cells. *Nature* **560**, 494-498, doi:10.1038/s41586-018-0414-6 (2018).

38 Neri, F. *et al.* Intragenic DNA methylation prevents spurious transcription initiation. *Nature* **543**, 72-77, doi:10.1038/nature21373 (2017).

39 VanDussen, K. L. *et al.* Notch signaling modulates proliferation and differentiation of intestinal crypt base columnar stem cells. *Development* **139**, 488-497, doi:10.1242/dev.070763 (2012).

40 Ishibashi, F. *et al.* Contribution of ATOH1(+) Cells to the Homeostasis, Repair, and Tumorigenesis of the Colonic Epithelium. *Stem Cell Reports* **10**, 27-42, doi:10.1016/j.stemcr.2017.11.006 (2018).

41 Chiacchiera, F., Rossi, A., Jammula, S., Zanotti, M. & Pasini, D. PRC2 preserves intestinal progenitors and restricts secretory lineage commitment. *EMBO J* **35**, 2301-2314, doi:10.15252/embj.201694550 (2016).

42 Frieda, K. L. *et al.* Synthetic recording and in situ readout of lineage information in single cells. *Nature* **541**, 107-111, doi:10.1038/nature20777 (2017).

43 Schmidt, F., Cherepkova, M. Y. & Platt, R. J. Transcriptional recording by CRISPR spacer acquisition from RNA. *Nature* **562**, 380-385, doi:10.1038/s41586-018-0569-1 (2018).

44 Trapnell, C. *et al.* The dynamics and regulators of cell fate decisions are revealed by pseudotemporal ordering of single cells. *Nat Biotechnol* **32**, 381-386, doi:10.1038/nbt.2859 (2014).

45 Haghverdi, L., Buttner, M., Wolf, F. A., Buettner, F. & Theis, F. J. Diffusion pseudotime robustly reconstructs lineage branching. *Nat Methods* **13**, 845-848, doi:10.1038/nmeth.3971 (2016).
