# Supplementary material for: Retrospective analysis of enhancer activity and transcriptome history
Source: Nat Biotechnol. 2023 Feb 23;41(11):1582–92. doi: 10.1038/s41587-023-01683-1 (PMC10635829; doi:10.1038/s41587-023-01683-1)
Supplement: Supplementary file 2 — Reporting Summary [file 41587_2023_1683_MOESM2_ESM.pdf]

nature research

Corresponding author(s): J Gribnau

Last updated by author(s): Jan 11, 2023

Reporting Summary

Nature Research wishes to improve the reproducibility of the work that we publish. This form provides structure for consistency and transparency in reporting. For further information on Nature Research policies, see our Editorial Policies and the Editorial Policy Checklist.

Statistics

For all statistical analyses, confirm that the following items are present in the figure legend, table legend, main text, or Methods section.

n/a	Confirmed	
☐	☒	The exact sample size (*n*) for each experimental group/condition, given as a discrete number and unit of measurement
☐	☒	A statement on whether measurements were taken from distinct samples or whether the same sample was measured repeatedly
☐	☒	The statistical test(s) used AND whether they are one- or two-sided<br>*Only common tests should be described solely by name; describe more complex techniques in the Methods section.*
☐	☒	A description of all covariates tested
☐	☒	A description of any assumptions or corrections, such as tests of normality and adjustment for multiple comparisons
☐	☒	A full description of the statistical parameters including central tendency (e.g. means) or other basic estimates (e.g. regression coefficient) AND variation (e.g. standard deviation) or associated estimates of uncertainty (e.g. confidence intervals)
☐	☒	For null hypothesis testing, the test statistic (e.g. *F*, *t*, *r*) with confidence intervals, effect sizes, degrees of freedom and *P* value noted<br>*Give P values as exact values whenever suitable.*
☒	☐	For Bayesian analysis, information on the choice of priors and Markov chain Monte Carlo settings
☒	☐	For hierarchical and complex designs, identification of the appropriate level for tests and full reporting of outcomes
☐	☒	Estimates of effect sizes (e.g. Cohen's *d*, Pearson's *r*), indicating how they were calculated

Our web collection on statistics for biologists contains articles on many of the points above.

Software and code

Policy information about availability of computer code

Data collection	Public data was retrieved from databases either directly through their website ENCODE portal (https://www.encodeproject.org) or using sra-tools v2.11.0.
Data analysis	In general, custom Python scripts are available upon request with exceptions to scripts concerning processing of raw MeD-seq data which are limited under licensing agreements between Erasmus MC and commercial partners. However, scripts regarding downstream analysis of MeD-seq data are available upon request. For the other analyses, used software packages are SAMtools v0.1.19, IGV v2.11.2, bbtools v37.62, bedtools v2.29.2, hisat2 v2.1.0, HTSeq v0.9.1, R software environment for statistical computing and graphics (version 3.4.0), EDASeq R package, EdgeR R package, chromVAR package, package motifmatchr, Monocle3 v0.2.0, UMAP v0.1.4, velocyto v0.17, scanpy v1.9.1, scvelo v0.2.4, deepTools, RTA3.4.4, v3.5.0, bowtie2 v2.4.1, MACS2 v2.2.7.1, Bcl2fastq v2.20, UMI-tools v1.1.2, Trim Galore v0.6.7 (wrapper of Cutadapt v1.18), bismark v0.23.1, BD FACSDiva software version 9.0.1 and FlowJo 10.7.2

For manuscripts utilizing custom algorithms or software that are central to the research but not yet described in published literature, software must be made available to editors and reviewers. We strongly encourage code deposition in a community repository (e.g. GitHub). See the Nature Research guidelines for submitting code & software for further information.

Data

Policy information about availability of data

All manuscripts must include a data availability statement. This statement should provide the following information, where applicable:

- Accession codes, unique identifiers, or web links for publicly available datasets
- A list of figures that have associated raw data
- A description of any restrictions on data availability

MeD-seq, WGBS and Cut&Tag sequencing data is deposited at NCBI with accession number PRJNA615329, RNA sequencing data is deposited at NCBI with accession

number PRJNA615329.
In addition the following datasets were downloaded and used for analysis. From the ENCODE portal (https://www.encodeproject.org): Mouse ES cell: ENCSR000CCC, ENCSR000CMW, ENCSR000CFO, ENCSR000CCD, ENCSR000CGN, ENCSR000CGO, ENCSR000CFZ, ENCSR779CZG, ENCSR392DGA, ENCSR000CGQ, ENCSR000CFN, ENCSR000CGR. Mouse intestine: ENCSR159RVN, ENCSR198ACZ, ENCSR311VKI, ENCSR642VYW, ENCSR389EYR, ENCSR483KOD, ENCSR000CEE, ENCSR079GOY from GEO: GSE83394 and from SRA using sra-tools v2.11.0.: SRX1560887, SRX1560888, 258 SRX1560889, SRX1560890, SRX3920113, SRX3920114, SRX3920117, SRX3920105, SRX3920106, SRX3920107, SRX3920108, SRX5023289, SRX5023290, SRX2339011, SRX2339012, SRX2339013, SRX2339022, SRX2339023, SRX2339024, SRX856956, SRX856957, SRX856959, SRX856960, SRX2339102, SRX2339103, SRX2339104, SRX2339111, SRX2339112, SRX2339113, SRX1817263, SRX1817257, SRX1817249, SRX1817250, SRX1817251, SRX1817253, SRX1817254, SRX1817252, SRX1817255. Single cell RNA-seq data: GSE92332 en GSE46980

Field-specific reporting

Please select the one below that is the best fit for your research. If you are not sure, read the appropriate sections before making your selection.

☒ Life sciences ☐ Behavioural & social sciences ☐ Ecological, evolutionary & environmental sciences

For a reference copy of the document with all sections, see nature.com/documents/nr-reporting-summary-flat.pdf

Life sciences study design

All studies must disclose on these points even when the disclosure is negative.

Sample size	No sample size calculations were made, variance of data points showed that aiming at commonly used triplicates was sufficient
Data exclusions	Data were only excluded if during sequencing insufficient reads could be generated and/or data complexity was too low.<br>Both are hallmarks of insufficient amounts of DNA or RNA provided for sequencing
Replication	Data were acquired as biological replicates, using independent triplicates. Replication of results was succesful, in addition results were confirmed using different methods.
Randomization	All sample conditions were randomly assigned.
Blinding	Blinding in mouse experiments was not possible because experimental and control groups were based on Doxycycline induction. For other experiments blinding was not relevant because it is never included in the type of experiments part of this study.

Reporting for specific materials, systems and methods

We require information from authors about some types of materials, experimental systems and methods used in many studies. Here, indicate whether each material, system or method listed is relevant to your study. If you are not sure if a list item applies to your research, read the appropriate section before selecting a response.

Materials & experimental systems

n/a	Involved in the study	
☐	☒	Antibodies
☐	☒	Eukaryotic cell lines
☒	☐	Palaeontology and archaeology
☐	☒	Animals and other organisms
☒	☐	Human research participants
☒	☐	Clinical data
☒	☐	Dual use research of concern

Methods

n/a	Involved in the study	
☒	☐	ChIP-seq
☐	☒	Flow cytometry
☒	☐	MRI-based neuroimaging

Antibodies

Antibodies used

SGLT1, Rabbit, IHC (1:200), Alomone Labs, AGT-031
SLC43A2, Rabbit, IHC (1:50), My Biosource, MBS9210948
SLC2A2/Glut2 Cy5, Rabbit, FC (5 µl per 1x10^6 cells), Bioss, bs-0351R-Cy5
EpCam 450, Rat, FC (2 µl per 1x10^6 cells), Invitrogen, 48-5791-82
CD31-BV421, Rat, FC (0.2 µl per 1x10^6 cells), BD Horizon, 563356
CD45-BV421, Rat, FC (0.2 µl per 1x10^6 cells), BD Horizon, 563890
TER119-BV421, Rat, FC 0.2 µl per 1x10^6 cells), BD Horizon, 563998
CD24-APC, Rat, FC (0.4 µl per 1x10^6 cells), Biolegend, 562349
CD117-PE (cKit), Rat, FC (0.3 µl per 1x10^6 cells), Biolegend, 105808
GNL3/nucleostemin, Goat, IHC (1:50), R&D Systems, AF1638
Nup54, Rabbit, IHC (1:50), Novus, NBP1-85899
CBX3, Rabbit, IHC (1:20), Proteintech, 11650-2AP
HCAM, Rat, IHC (1:50), Santa Cruz, sc-18849

H2AK119Ac, Rabbit, IHC (1:500), gift Zu-Wen Sun
H3K9me2, Rabbit, IHC (1:100), Upstate, 07-212
H3K9me3, Rabbit, IHC (1:200), Diagenode, cs-056-050
Histone H2A.Z, Rabbit, IHC (1:500), CUT&Tag (1:100), Abcam, ab4174
Acetyl histone H2A.Z, Rabbit, IHC (1:500), CUT&Tag (1:100), Merck ABE1363
H3K27me3, Rabbit, IHC (1:100), Cell Signaling Technology, 9733
rabbit α-mouse antibody, IHC (1:100), Abcam, ab46540
Mouse Ring1B clone # 3, (1:2), Atsuta, T., Fujimura, Y., Moriya, H., Vidal, M., Akasaka, T. and Koseki, H. (2001). Production of monoclonal antibodies against mammalian Ring1B proteins. Hybridoma)
Goat anti rat Alexa488, IHC (1:500), Invitrogen, A-11006
Goat anti rabbit Alexa488, IHC (1:500), Invitrogen, A-11008
Streptavidin Alexa488, IHC (1:200), Invitrogen, s-32354
Goat anti rat Alexa546, IHC (1:500), Invitrogen, A-11081
Goat anti rabbit Alexa546, IHC (1:500), Invitrogen, A-11010
Donkey anti rabbit 546, IHC (1:500), Invitrogen, A-10040
Donkey anti goat Alexa555, IHC (1:500), Invitrogen, A-21432
Rabbit anti rat biotinylated, IHC (1:200), Dako, E0468
TSA Biotin Systems, Perkin Elmer, NEL700A
Rabbit anti DCM, Rabbit, WB (1:1000), Cusabio, CSB-PA365131XA01ENV
Rabbit anti PolR2B, Rabbit, WB (1:1000), ThermoFisher, PA5-30122
beta-Actin-Peroxidase, clone AC-15, Mouse, WB(1:7500), Sigma-Aldrich, A3854
HRP, goat anti Rabbit, WB(1:5000), Sigma-Aldrich, A6154

Validation

Anti-SGLT1: https://www.alomone.com/p/anti-sglt1-extracellular/AGT-031
Validated through results on supplier's website and linked references on that website.
Anti-SLC43A2: https://www.mybiosource.com/pathway/124935
Validated through results on supplier's website and linked references on that website.
Anti-SLC2A2/Glut2: https://www.biossusa.com/products/bs-0351r-cy5
Validated through results on supplier's website and linked references on that website.
Anti-EpCam: https://www.thermofisher.com/antibody/product/CD326-EpCAM-Antibody-clone-G8-8-Monoclonal/48-5791-82
Validated through results on supplier's website and linked references on that website.
Anti-CD31: https://www.citeab.com/antibodies/2407732-563356-bd-horizon-bv421-rat-anti-mouse-cd31
Validated through results on supplier's website and linked references on that website.
Anti-CD45: https://www.bdbiosciences.com/en-au/products/reagents/flow-cytometry-reagents/research-reagents/single-color-antibodies-ruo/bv421-rat-anti-mouse-cd45.563890
Validated through results on supplier's website and linked references on that website.
Anti-TER119: https://www.bdbiosciences.com/en-us/products/reagents/flow-cytometry-reagents/research-reagents/single-color-antibodies-ruo/bv605-rat-anti-mouse-ter-119-erythroid-cells.563998
Validated through results on supplier's website and linked references on that website.
Anti-CD24: https://www.biolegend.com/fr-ch/products/apc-anti-mouse-cd24-antibody-2937
Validated through results on supplier's website and linked references on that website.
Anti-CD117: https://www.biolegend.com/nl-be/products/pe-anti-mouse-cd117-c-kit-antibody-75
Validated through results on supplier's website and linked references on that website.
Anti-GNL3: https://www.biocompare.com/9776-Antibodies/121935-HumanMouseRat-Nucleostemin-Affinity-Purified-PAb/
Validated through results on supplier's website and linked references on that website.
Anti-Nup54: https://www.novusbio.com/products/nup54-antibody_nbp1-85899
Validated through results on supplier's website and linked references on that website.
Anti-CBX3: https://www.ptglab.com/products/CBX3-Antibody-11650-2-AP.htm
Validated through results on supplier's website and linked references on that website.
Anti-HCAM: https://www.scbt.com/p/hcam-antibody-im7
Validated through results on supplier's website and linked references on that website.
Anti-H2AK119Ac: https://pubmed.ncbi.nlm.nih.gov/?term=H2AK119Ac%20Zu-Wen%20Sun&sort=date
From Zu-Wen Sun, used in multiple articles available on pubmed
Anti-H3K9me2: https://www.sigmaaldrich.com/NL/en/product/mm/07212
Validated through results on supplier's website and linked references on that website.
Anti-H3K9me3: https://www.diagenode.com/en/p/h3k9me3-polyclonal-antibody-classic-50-ug
Validated through results on supplier's website and linked references on that website.
Anti-H2A.Z: https://www.abcam.com/histone-h2az-antibody-chip-grade-ab4174.html
Validated through results on supplier's website and linked references on that website.
Anti-H2A.Zac: https://www.merckmillipore.com/NL/en/product/Anti-acetyl-Histone-H2A.Z-Lys-5-7-11-Antibody,MM_NF-ABE1363
Validated through results on supplier's website and linked references on that website.
Anti-H3K27me3: https://www.cellsignal.com/products/primary-antibodies/tri-methyl-histone-h3-lys27-c36b11-rabbit-mab/9733
Validated through results on supplier's website and linked references on that website.
Anti-Ring1B: clone # 3 Atsuta, T., Fujimura, Y., Moriya, H., Vidal, M., Akasaka, T. and Koseki, H. (2001). Production of monoclonal antibodies against mammalian Ring1B proteins.
Anti-DCM: https://www.cusabio.com/Custom-Antibodies/DCM-Antibody-12831372.html
Validated through results on supplier's website and linked references on that website.
Anti-PolR2B: https://www.thermofisher.com/antibody/product/POLR2B-Antibody-Polyclonal/PA5-30122
Validated through results on supplier's website and linked references on that website.
Anti-beta-Actin-Peroxidase: https://www.sigmaaldrich.com/NL/en/product/sigma/a3854
Validated through results on supplier's website and linked references on that website.

Eukaryotic cell lines

Policy information about cell lines

Cell line source(s)	Generated in our lab
Authentication	Authentication was done using PCR
Mycoplasma contamination	All cell lines used were tested for Mycoplasma and confirmed to be negative
Commonly misidentified lines (See ICLAC register)	*Name any commonly misidentified cell lines used in the study and provide a rationale for their use.*

Animals and other organisms

Policy information about studies involving animals; ARRIVE guidelines recommended for reporting animal research

Laboratory animals	Mus Musculus (C57BL/6), both female and male, adult (>8 weeks old)
Wild animals	None
Field-collected samples	None
Ethics oversight	All animal experiments were approved by the Dutch Central Committee on the Ethics of Animal Experiments (AVD10100202115681)

Note that full information on the approval of the study protocol must also be provided in the manuscript.

Flow Cytometry

Plots

Confirm that:

☒ The axis labels state the marker and fluorochrome used (e.g. CD4-FITC).

☒ The axis scales are clearly visible. Include numbers along axes only for bottom left plot of group (a 'group' is an analysis of identical markers).

☒ All plots are contour plots with outliers or pseudocolor plots.

☒ A numerical value for number of cells or percentage (with statistics) is provided.

Methodology

Sample preparation	Cells were incubated with antibodies (Epcam (Cd326, eFluor 450 eBioscience™ from ThermoFisher, Catalog # 48-5791-82) and SLC2A2 (GLUT-2, cy5 from Bioss, Catalog # bs-0351R-Cy5) for 45 minutes at 4oC, protected from light, and washed twice in 1 mL of cold PBS. After final centrifugation (5 minutes at 200 g, 4oC) cells were resupended in 1 mL cold PBS and filtered through a 40 uM cell strainer before proceeding with FACS-sorting. Cells stained for Cd326 and Glut-2 were FACS sorted using a BD FACS Aria II and double positive cell populations were isolated collecting >10.000 enterocytes per time point.
Instrument	BD FACS Aria II
Software	BD FACSDiva software, version 9.0.1 for sorting<br>FlowJo 10.7.2 for analysis
Cell population abundance	Post-sort abundance was determined using RNA-seq and validated using published scRNA-seq
Gating strategy	Live cells were selected based on SSC-A and FSC-A, single cells on FSC-A and FCS-W. Positive populations were selected based on isotype negative controls. Double positive populations were distinct from the negative.<br>Box can be ticked. There is a figure in the supp info about sorting.

☒ Tick this box to confirm that a figure exemplifying the gating strategy is provided in the Supplementary Information.
